# New Copper (II) Complexes Based on 1,4-Disubstituted-1,2,3-Triazole Ligands with Promising Antileishmanial Activity

**DOI:** 10.3390/pharmaceutics18010064

**Published:** 2026-01-04

**Authors:** João P. C. Nascimento, Natali L. Faganello, Karolina F. Freitas, Leandro M. C. Pinto, Amarith R. das Neves, Diego B. Carvalho, Carla C. P. Arruda, Sidnei M. Silva, Rita C. F. Almeida, Amilcar M. Júnior, Davi F. Back, Lucas Pizzuti, Sumbal Saba, Jamal Rafique, Adriano C. M. Baroni, Gleison A. Casagrande

**Affiliations:** 1 Grupo de Pesquisa em Síntese e Caracterização Molecular do Mato Grosso do Sul, Instituto de Química—Universidade Federal de Mato Grosso do Sul—UFMS (Laboratório 2), Av. Senador Filinto Muller 1555, Campo Grande 79074-460, MS, Brazil; jpcn2020@hotmail.com (J.P.C.N.); leandro.pinto@ufms.br (L.M.C.P.);; 2 Laboratório de Síntese e Química Medicinal, LASQUIM, Universidade Federal do Mato Grosso do Sul, Campo Grande 79070-900, MS, Brazil; 3 Laboratório de Parasitologia Humana, Instituto de Biociências, Universidade Federal de Mato Grossso do Sul—UFMS, Campo Grande 79070-900, MS, Brazil; 4 Química, Campus-Sede, Universidade de Caxias do Sul—UCS, Caxias do Sul 95070-560, RS, Brazil; 5 Instituto de Química, Universidade Federal do Rio Grande do Norte, Av. Senador Salgado Filho, s/n, Natal 59078-970, RN, Brazil; 6 Laboratório de Materiais Inorgânicos, Departamento de Química, Universidade Federal de Santa Maria—UFSM, Av. Roraima 1000, Santa Maria 97105-900, RS, Brazil; 7 Grupo de Pesquisa em Síntese e Caracterização Molecular do Mato Grosso do Sul, Universidade Federal da Grande Dourados, Dourados 79825-070, MS, Brazil; lucaspizzuti@ufgd.edu.br; 8 LabSO, Instituto de Química, Universidade Federal de Goiás—UFG, Goiânia 74690-900, GO, Brazil; sumbalsaba@ufg.br

**Keywords:** copper (II) complexes, triazole, ligand, antileishmanial activity, metallopharmaceutical, bioactive molecules

## Abstract

**Background/Objectives:** Leishmaniasis constitutes one of the most fatal parasitic diseases globally, adversely impacting the health of individuals residing in both intertropical and temperate zones. In these geographical areas, the administration of treatment is often inconsistent and largely ineffective with the available pharmaceuticals, as these exhibit more pronounced side effects than the therapeutic advantages they purport to provide. **Methods**: Consequently, the current investigation seeks to engage in molecular modeling of novel pharmacological candidates incorporating 1,2,3 disubstituted triazole moieties, coordinated with Cu^II^ metal centers, in pursuit of promising bioactive properties. **Results**: Two complexes were prepared and X-ray analysis revealed a comparable structural configuration surrounding the copper (II) atom. The planar square coordination geometry was elucidated through the assessment of the $\tau_{4}=0$ (tau four) parameters. The comprehensive characterization encompasses HRMS-ESI (+), NMR, elemental analyses, mid-infrared, and UV-vis spectroscopic techniques. Time-dependent density functional theory (TD-DFT) analyses will substantiate the findings obtained through UV-vis spectroscopy. Crucially, the biological assays against *Leishmania* (*L.*) *amazonensis* revealed that Complex 1 exhibited outstanding potency against the intracellular amastigote form, demonstrating a half-maximal inhibitory concentration (IC_50_) of 0.4 µM. This activity was 6-fold higher than that of amphotericin B (IC_50_ = 2.5 µM) and 33-fold higher than pentamidine (IC_50_ = 13.3 µM). Furthermore, Complex 1 showed a promising selectivity index (SI = 9.7) against amastigotes, surpassing the reference drugs and meeting the criteria for a lead compound. While less active on promastigotes, both complexes demonstrated high stability in DMSO solution, a prerequisite for biological testing. **Conclusions**: These results unequivocally identify Complex 1 as a highly promising candidate for the development of new antileishmanial therapies, warranting further in vivo studies.

## 1. Introduction

Leishmaniasis represents a complex of diseases caused by *Leishmania* parasites. It is a neglected tropical disease (NTD) with prevalence in the Americas, East Africa, North Africa, and West and Southeast Asia. The disease is mostly present in tropical areas and among vulnerable communities [1,2,3]. With nearly 1 billion people living in areas where Leishmaniasis is endemic and up to 1 million new cases occurring annually, the global burden of this disease is immense. This stark reality underscores the critical and urgent need for the development of new, safer, and more effective treatments for all forms of the disease [1,3].

The clinical forms of the disease are cutaneous leishmaniasis (CL), mucocutaneous leishmaniasis (MCL), and visceral leishmaniasis (VL). Cutaneous leishmaniasis (CL) is the most common clinical form of the disease prevalent in Latin American countries [1,2].

The biological cycle of *Leishmania* parasites is complex and multifaceted, with more than fifty different vector species and approximately twenty-two species of *Leishmania* involved in the human pathogenic cycle. In Brazil, CL is mainly caused by *Leishmania* (*Leishmania*) *amazonensis* parasites [3].

The treatment of leishmaniasis relies primarily on pentavalent antimonials, such as *N*-methylglucamine antimoniate (Glucantime^®^) and sodium stibogluconate (Stiboson^®^). However, these drugs require prolonged administration, during which adverse effects often outweigh their therapeutic benefits. Similar challenges are observed with second-line drugs, including amphotericin B, pentamidine, paromomycin, and miltefosine, where the main limitations involve high cost, suboptimal efficacy, and the development of parasites resistance [4,5,6,7,8].

Compounds bearing triazole nucleus and their derivatives have demonstrated significant potential in medicinal chemistry, as evidenced by their wide range of biological activities [9]. Their applications include anticancer [10,11], anticonvulsant [12,13,14], antimicrobial [15], antiviral [16] antitubercular [17], antidiabetic [18], anti-inflammatory [19], antiproliferative [20], antioxidant [21], antimalarial [22], and leishmanicidal activities [23,24,25], highlighting the versatility and therapeutic relevance of these compounds across diverse biomedical contexts.

Copper plays a crucial role in biological systems, as many enzymes and living organisms depend on copper-containing compounds for energy production. Oxygen transport in various organisms, including mollusks and arthropods, is facilitated by the copper protein hemocyanin, which differs from hemoglobin in performing this function extracellularly. Biological mechanisms involving copper are tightly regulated, as fluctuations in copper levels may lead to disorders such as Wilson’s disease (characterized by copper accumulation) and Menkes disease (resulting from copper deficiency) [26].

Copper complexes are recognized as versatile biocidal agents due to their ability to disrupt cellular membranes and proteins, inhibit reduced glutathione, and form stable interactions with nucleic acids. The coordination of copper with polycyclic organic ligands has been proposed as a strategy to mitigate the reactivity of unbound copper while enabling targeted delivery to specific organisms or biological sites [26,27]. These organic ligands also enhance cellular uptake and improve the biological properties of the complexes. Copper complexes showed inhibitory effects with IC_50_ values in the micromolar (μM) range across several cancer cell lines. In addition, evidence suggests that they may interfere in the cell cycle progression, particularly during mitosis [28,29,30]. This interference is often attributed to the ability of planar, aromatic heterocyclic systems, like the triazole nucleus, to interact with biological targets involved in mitotic regulation, such as tubulin. By potentially inhibiting tubulin polymerization, these compounds can disrupt spindle formation, leading to mitotic arrest and, consequently, apoptosis in rapidly dividing cells.

In this context, copper complexes are considered promising antileishmanial agents, becoming the focus of increasing investigation in recent years [31]. For example, complexes incorporating triazolopyrimidine ligands coordinated with copper exhibited IC_50_ values ranging from 20 to 60 μM after 72 h of exposure against two *Leishmania* species (*L. infantum* and *L. braziliensis*) at the promastigote stage [32]. In another study, a triazolopyrimidine derivative demonstrated efficacy of approximately 20 μM against *L. infantum* and *L. braziliensis* in the promastigote stage [33].

The development of novel metallodrugs based on copper coordination compounds with triazole ligands has emerged as an active and prolific field of research, given the strong activity of the free ligand and the expectation that newly designed compounds may surpass both the efficacy and toxicity limitations of the current antileishmanial drugs.

As part of our wider research interest in bioactive compounds [34,35,36,37,38,39], and aiming to advance in the drug discovery of new antileishmanial agents, herein we report the synthesis, structural characterization, and in vitro antileishmanial evaluation against *L.* (*L.*) *amazonensis* of new copper (II) coordination compounds with 1,4-diaryl-1,2,3-triazole ligands previously studied against *L.* (*L.*) *amazonensis* [24,25].

## 2. Materials and Methods

### 2.1. Chemistry

Detailed information about chemicals, instrumentation, methodology and calculations are described in the Supporting Information of this paper. Synthesis of ligands followed the methodology described by us previously [24,25].

### 2.2. Synthesis of the Complexes

**Scheme 1:**
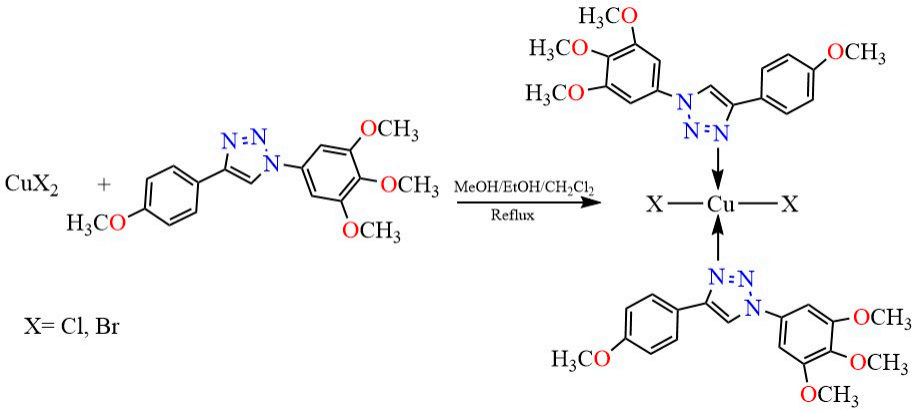
Synthesis of the complexes.

The complexes were obtained through the reaction between Cu^II^ chloride 0.034 g (0.2 mmol) and Cu^II^ bromide 0.0513 g (0.23 mmol), with triazole ligand 0.068 g (0.2 mmol), in a 1:1 ratio. A mixture of MeOH/EtOH/CH_2_Cl_2_ was used in the ratio 2:1:3 in milliliters (mL), under stirring and reflux for 90 min (Scheme 1). Complexes **1** and **2** were obtained in the form of brown crystals in the mother solution after 1 week of slow evaporation at room temperature Figures S1 and S2.

Ligand **L1**: was obtained as a white solid; melting point 155 °C; Elemental analyses

(CHN %) Calc. C 63.33, H 5.61, N 12.31 (%); Found C 63.12, H 5.65, N 12,37; IR: 3141 **ν(C-H)_trz_**, 2941-2831 **ν(C-H)**, 1598 **ν(C=N)**, 1510 **ν(C=C)**, 1230 **ν(C-O-CH_3_)**, 1303 **ν(N-N)**; ^1^H NMR (500 MHz, DMSO-d_6_) *δ* (ppm) 9. 17 (s, 1H), 7.86 (d, *J* = 8.9 Hz, 2H), 7.26 (s, 2H), 7.08 (d, *J* = 8,9 Hz, 2H), 3.91 (s, 6H), 3.82 (s, 3H), 3.73 (s, 3H). ^13^C NMR (126 MHz, DMSO-d_6_) *δ* 159.30, 153.55, 147.12, 137.31, 132.58, 126.68, 122.82, 118.85, 114.43, 97.88, 60.23, 56.30, 55.21.

Complex **1**: Yield: 57% in crystals, melting point 185 °C; Elemental Analysis CHN theoretical: C 52,91%, H 4,69% e N 10,28%. CHN experimental C 52,90%, H 4,66% e N 10,27%; IR 3132-3110 **ν(C-H)_trz_**, 2941-2831 **ν(C-H)**, 1614 **ν(C=N)**, 1502 **ν(C=C)**, 1247 **ν(C-O-CH_3_)**, 1319 **ν(N-N)**; HRMS (ESI+) *m*/*z* 342.1447 (C_18_H_20_N_3_O_4_^+^), 713.7386 [C_72_H_76_CuN_12_O_16_]^+2^, 745.2072 [C_36_H_38_CuN_6_O_8_]^+^, 780.1753 [C_36_H_18_ClCuN_6_O_8_]^+^, 1086.3467 [C_54_H_57_CuN_9_O_12_]^+^, 1121.3155 [C_54_H_57_ClCuN_9_O_12_]^+^.

Complex **2**: Yield 60% in crystals, melting point 175 °C; Elemental Analysis CHN theoretical: C 47,72%, H 4,23% e N 9,28%. CHN experimental C 47,75%, H 4,25% e N 9,26%; IR 3128-3109 **ν(C-H)_trz_**, 2933-2831 **ν(C-H)**, 1612 **ν(C=N)**, 1500 **ν(C=C)**, 1249 **ν(C-O-CH_3_)**, 1317 **ν(N-N)**; HRMS (ESI+) *m*/*z* 342.1034 [C_18_H_20_N_3_O_4_]^+^, 713.7269 [C_72_H_76_CuN_12_O_16_]^+2^, 745.2055 [C_36_H_18_CuN_6_O_8_]^+^, 824.1513 [C_36_H_38_BrCuN_6_O_8_]^+^, 1086.5101 [C_54_H_57_CuN_9_O_12_]^+^, 1165.4816 [C_54_H_57_BrCuN_9_O_12_]^+^.

### 2.3. Biological Activity

#### 2.3.1. Antileishmanial Assays

The complexes were initially solubilized in dimethyl sulfoxide (DMSO, Sigma-Aldrich^®^, Cajamar, Brazil) at a concentration of 5 mg/mL. A fresh stock solution was prepared immediately prior to the assays and subsequently diluted in the appropriate culture medium containing 1% DMSO (*v*/*v*). Serial dilutions were performed to achieve the desired range of working concentrations.

#### 2.3.2. Animals and Parasites

Female BALB/c mice (4–5 weeks old, approximately 30 g) were sourced from the Central Animal Facility at the Federal University of Mato Grosso do Sul (UFMS, Campo Grande, MS, Brazil). Animals were housed in mini-isolators connected to a ventilated rack system (Alesco^®^, Monte Mor, Brazil), maintained under specific pathogen-free (SPF) conditions at a controlled temperature of 25 ± 1 °C, with a 12 h light/dark cycle, and provided with standard rodent chow (Nuvital^®^, Canguiri Colombo, Brazil) and water ad libitum. All experimental procedures were conducted in compliance with the ethical guidelines approved by the Institutional Animal Care and Use Committee (CEUA/UFMS; protocol number 1.252/2022).

*Leishmania* (*Leishmania*) *amazonensis* (strain IFLA/BR/1967/PH8) was routinely isolated from lesion biopsies of infected BALB/c mice and propagated in vitro as promastigotes at 26 °C. Parasites were cultured in Schneider’s Insect Medium (Sigma-Aldrich^®^, Cajamar, Brazil) supplemented with 20% heat-inactivated fetal bovine serum (FBS, Sigma-Aldrich^®^, Cajamar, Brazil), penicillin (10,000 U/mL), and streptomycin (10 mg/mL) (Sigma-Aldrich^®^, Cajamar, Brazil), with a maximum of ten serial in vitro passages used for experimental procedures.

#### 2.3.3. Peritoneal Macrophages

A total volume of 10 mL of cold RPMI 1640 medium (Sigma-Aldrich^®^), supplemented with 10,000 U/mL penicillin and 10 mg/mL streptomycin (Sigma-Aldrich^®^), was injected into the peritoneal cavity of the mice. After gentle abdominal massage, peritoneal macrophages were released and harvested. Cell counts were performed using a Neubauer chamber, following exclusion of non-viable cells via trypan blue staining (Sigma-Aldrich^®^) [34].

#### 2.3.4. Antipromastigote Assay

The complexes were evaluated in quintuplicate at final concentrations ranging from 50.0 µg/mL to 0.78 µg/mL. In 96-well microplates, a solution of Schneider’s Insect Medium (Sigma-Aldrich^®^) supplemented with promastigote forms of *Leishmania* (*L.*) *amazonensis* (2 × 10^5^ parasites per well) was added, and the plates were incubated at 26 °C for 72 h in a Biochemical Oxygen Demand (BOD) incubator (Cienlab^®^, Campinas, Brazil). Cell viability was assessed using the MTT assay (5 mg/mL, Thiazolyl Blue Tetrazolium Bromide, Sigma-Aldrich^®^). The microplates were incubated at 37 °C in a 5% CO_2_ atmosphere. After 4 h of incubation, dimethyl sulfoxide (DMSO, Sigma-Aldrich^®^) was added to solvate the formazan crystals. Pentamidine (12.5 µg/mL–0.19 µg/mL) and amphotericin B (1.0 µg/mL–0.015 µg/mL) were used as positive controls. The negative control consisted of DMSO in Schneider’s Insect Medium (Sigma-Aldrich^®^). The optical density (OD) was measured using a microplate spectrophotometer (Molecular Devices, SpectraMax Plus, Sunnyvale, USA) at 570 nm. The IC_50_ values were calculated by performing a non-linear dose–response regression analysis using GraphPad Prism 8.0 software (GraphPad Software^®^, San Diego, CA, USA) [35].

#### 2.3.5. Treatment of Infected Macrophages

Peritoneal macrophages (1 × 10^6^ cells/well) in RPMI 1640 medium (Sigma-Aldrich^®^) supplemented with 10% of FBS (Sigma-Aldrich^®^) were added to 24-well plates containing round glass coverslips (13mm) in the bottom, in six replicates. Plates were incubated for one hour at 37 °C in an atmosphere of 5% CO_2_ for adhesion. After, the adhered macrophages were infected with promastigote forms of *L.* (*L.*) *amazonensis* in the stationary growth phase (4 × 10^6^ parasites/mL) and incubated at 35 °C/5% CO_2._ After four hours, the infected cells were treated for 24 h with complexes (6.25–50 μg/mL). Pentamidine and amphotericin B (Sigma-Aldrich^®^; 6.25–50 μg/mL) were used as the reference drugs. Untreated cells were used as a negative control. After treatment, the supernatant was removed and the cells were washed with PBS (Sigma-Aldrich^®^), fixed with Bouin’s solution (Sigma-Aldrich^®^), and stained with Giemsa (Sigma-Aldrich^®^) diluted in 1:10 proportion in distilled water. The total number of amastigotes was determined by counting 200 cells per coverslip in six replicates, in an optical microscope. The half-maximal inhibitory concentration (IC_50_) was calculated using a non-linear regression curve with GraphPad Prism 8.0 software (GraphPad Software^®^, San Diego, CA, USA) [34].

#### 2.3.6. Cytotoxicity Evaluation

BALB/c peritoneal macrophages (1 × 10^6^ cells/well) were suspended in RPMI 1640 medium (Sigma-Aldrich^®^) supplemented with 10% fetal bovine serum (FBS, Sigma-Aldrich^®^) and seeded into 96-well plates in quintuplicate, following cell counting in a Neubauer chamber. The plates were incubated for 1 h at 37 °C in a 5% CO_2_ atmosphere to allow cell adhesion. After adhesion, the macrophages were exposed to test complexes at concentrations ranging from 250.0 µg/mL to 3.9 µg/mL and incubated at 35 °C in 5% CO_2_ for 48 h. Cell viability was evaluated using the MTT assay. Doxorubicin (Sigma-Aldrich^®^) was used as a positive control for cell death, applied at concentrations of 250.0–3.9 µg/mL. Dimethyl sulfoxide (DMSO, Sigma-Aldrich^®^) served as a negative control at the concentration required to solubilize the highest concentration of the test compounds, ensuring no interference with cell viability. Amphotericin B (250.0 µg/mL–3.9 µg/mL) was used as the reference antileishmanial drug. The 50% cytotoxic concentration (CC_50_) was determined by fitting a sigmoidal dose–response curve using GraphPad Prism 8.0 software (GraphPad Software^®^, San Diego, CA, USA).

## 3. Results and Discussions

### 3.1. Crystalline Structure Description

**Figure 1:**
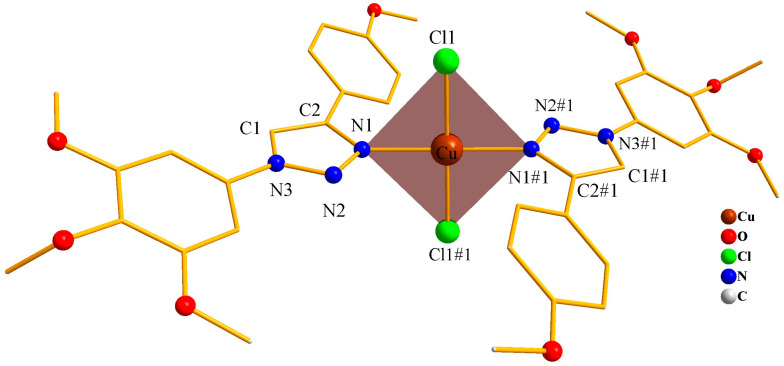
Series of synthesized TSCs evaluated in this work.

Since the two prepared complexes are related by an isostructural crystallographic relationship within the space group *P*$\bar{1}$, this report addresses their main structural features collectively. Detailed information on crystallographic parameters, including bond angles and interatomic distances, is provided in the (Supplementary Information Tables S1–S3). The molecular and crystal architecture of compound 1 is illustrated in Figure 1.

Figure 1 and Figure S3 show that complexes **1** and **2**, respectively, are formed by two molecules of ligand L1 coordinated in a monodentate manner to the copper (II) atom via the N1 atom, along with two halide atoms from the metal salt, completing the coordination sphere.

The square planar geometry around the copper atom was assessed using the trigonalization parameter (τ_4_) proposed by Lei Yang, Douglas R. Powell, and Robert P. Houser [40], which is inspired by the trigonality parameter introduced by Addison and Reedijk [41]. This parameter provides a quantitative measure for four-coordinate complexes, allowing for a systematic assessment of deviations from ideal square planar geometry and facilitating direct comparison between different coordination environments. The τ_4_ parameter was calculated by using the following equation:

$$\tau_{4}=\;\frac{360-\;(\alpha +\beta )}{141{}^{\circ}}$$

Here, α and β represent the two largest bond angles around the coordination center. For a τ_4_ value close to 1.00, the coordination geometry approaches a tetrahedral arrangement, whereas a τ_4_ value of 0 corresponds to an ideal square planar geometry. τ_4_ values between 0 and 1.00 indicate intermediate geometries, which may represent distortions of the two extremes or other geometries, such as a trigonal pyramidal arrangement.

**Figure 2:**
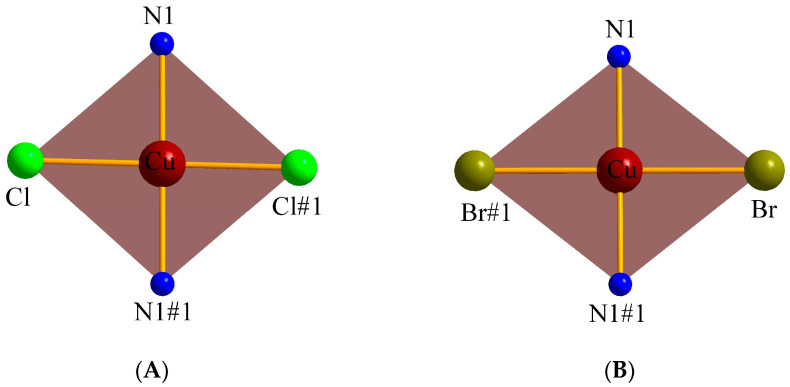
Coordination polyhedron representation: (**A**) the coordination sphere of complex **1** (**A**) and (**B**) the coordination sphere of complex **2**.

For the synthesized complexes, the two largest bond angles at the coordination center are 180° (Tables S2 and S3), measured between the halide atoms and the two nitrogen atoms of the ligands. Consequently, the τ_4_ value for both structures are 0, indicating an ideal square planar geometry, as also illustrated by the coordination polyhedron depicted in Figure 2.

The bond lengths around the coordination sphere include Cu (1)-Cl (1) and Cu (1)-Cl (#1) of 2.2411 Å, as well as Cu-N (1) and Cu-N (#1) of 1.9970 Å for complex 1. For complex 2, the corresponding bond lengths are Cu-Br (1) and Cu-Br (#1) of 2.4314 Å, and Cu-N (1) and Cu-N (#1) of 1.9503 Å. These values are consistent with those reported for similar complexes in the literature [42,43].

Figures S4 and S5 show the projection of the contents within the unit cell for complexes 1 and 2, respectively, highlighting the symmetry elements associated with the space group *P*1$\bar{1}$. Figures S6 and S7, along with Table S4, present the hydrogen bonding interactions observed. Complex **1** arranges the crystal structure in a dimeric form via intermolecular hydrogen bonds C (1)-H (1)…Cl#2 (2.93 Å) and C (16)-H (16B)…Cl#3 (2.95 Å).

For complex **2**, a similar dimeric arrangement is observed, involving the hydrogen bond C (1)-H (1)…Br#2 (2.85 Å). However, due to crystal packing, the hydrogen bond involving the -OCH_3_ group occurs at a longer distance of 3.00 Å, which falls outside the range considered in this study. The hydrogen bond lengths observed for both complexes are consistent with values reported for similar complexes in the literature [44,45].

### 3.2. Spectroscopic in the Mid-Infrared Region and Spectrometric Remarks

The main vibrational modes involving the free ligands and the prepared complexes are summarized in Table 1.

The observed vibrational modes were assigned and compared with those of similar compounds reported in the literature [45,46,47]. In general, the IR spectra of the complexes displayed notable differences compared to the free ligand (Figure S8), particularly in the regions associated with ν(C=N) stretching vibrations, which shifted from 1562 cm^−1^ in the free ligand to 1581 and 1579 cm^−1^ for complexes 1 and 2, respectively.

Regarding the free ligand, a prominent band at 3141 cm^−1^, corresponding to the ν(C–H) stretching of the triazole ring, was observed. This band, characteristic of the triazole moiety, is shifted in the complexes and exhibits splitting into two peaks, likely reflecting the formation of hydrogen bonding in the solid state, as confirmed by X-ray diffraction (XRD) analysis. Other stretching vibrations also show significant shifts in the spectra of the complexes (Figures S9 and S10).

The main fragments HRMS (ESI+) were detected and attributed as shown in Figures S11–S19 which show the spectra of complexes **1** and **2**. Both complexes revealed a general trend fragmentation pattern for both prepared Cu^II^ complexes. In general, the complexes undergo loss of the two halide ions, resulting in mass peaks at *m*/*z* 780.1753 and 745.2072, corresponding to the partial [C_36_H_18_ClCuN_6_O_8_]^+^ or complete [C_36_H_38_CuN_6_O_8_]^+^ loss of halides. These two species are also observed for complex 2.

Further fragmentation of the complexes is evidenced by the detection of the free ligand fragment at *m*/*z* 342.1447. For the peaks corresponding to partial halide loss, the observed isotopic pattern is consistent with the natural isotopes of copper (^63^Cu and ^65^Cu) as shown in Table S5.

### 3.3. Spectroscopic Remarks in the Ultraviolet-Visible (UV-Vis) Region

The triazole ligand appears as a white solid compound and exhibits a strong absorption band in the UV-Vis spectrum between 250 and 300 nm [45]. These bands are attributed to π → π* or n → π* electronic transitions arising from the aromatic rings present in the ligand.

**Figure 3:**
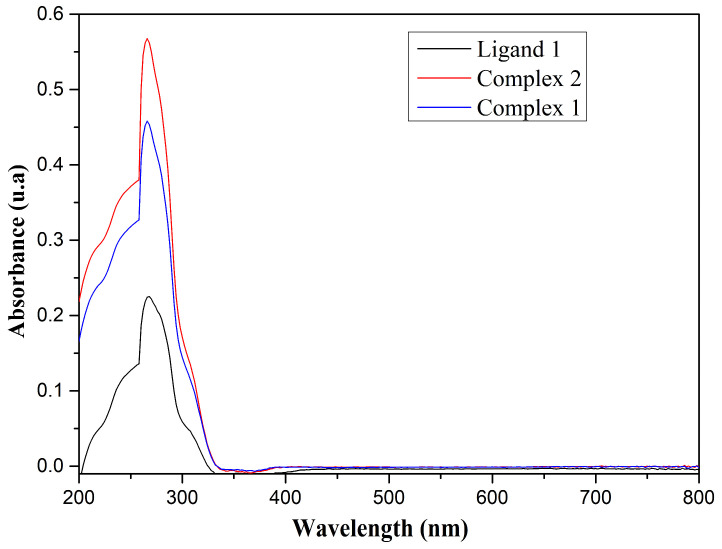
Absorption spectra of ligands and complexes 1 and 2 measured at 298 K in DMSO solution (≈1.0 × 10^−5^ M).

The absorption profiles of the complexes are similar to that of the free ligand, with a single absorption band appearing in the same region at the studied concentration (Figure 3). Notably, the complexes exhibit a hyperchromic effect (increased absorption intensity) in the UV-Vis spectrum compared to the free ligand, suggesting coordination of the ligand to the Cu^II^ metal center. The hyperchromic effect is also related to the appearance of XLCT, LMCT and IL (two ligands coordinated) electronic transitions according to the DFT calculations.

To further investigate the absorption processes involved in the electronic transitions during the formation of the coordination compounds, TD-DFT calculations were performed. Based on the known crystal structure of the complexes and considering their isostructural nature, the calculations were carried out by using dichloromethane as a non-coordinating solvent in vacuo. (Figure S20, Table S6).

**Figure 4:**
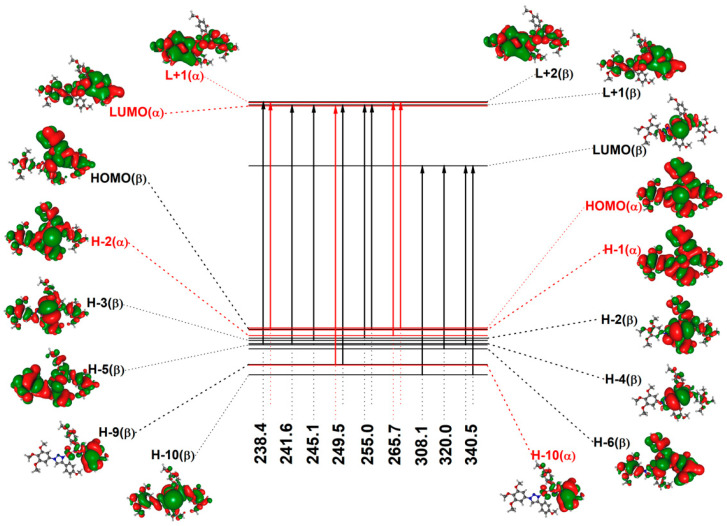
Frontier orbitals of complex 1 as per DFT calculations. DFT calculations drew on molecular structure (bond lengths and angles) determined by X-ray analysis.

Considering the transitions with the highest oscillator strengths (Figure 4), the S7 state, corresponding to the HOMO–3 (β) → LUMO + 1 (β) transition, can be assigned to an intraligand (IL) transition with additional halide-to-ligand charge transfer (XLCT) character.

Transitions S1 (HOMO–5 (β) → LUMO (β) and HOMO–10 (β) → LUMO (β)), S2 (HOMO–6 (β) → LUMO (β)), and S3 (HOMO–10 (β) → LUMO (β)) contribute to the single absorption band observed near 250 nm. These transitions are associated with ligand-to-metal charge transfer (LMCT).

**Figure 5:**
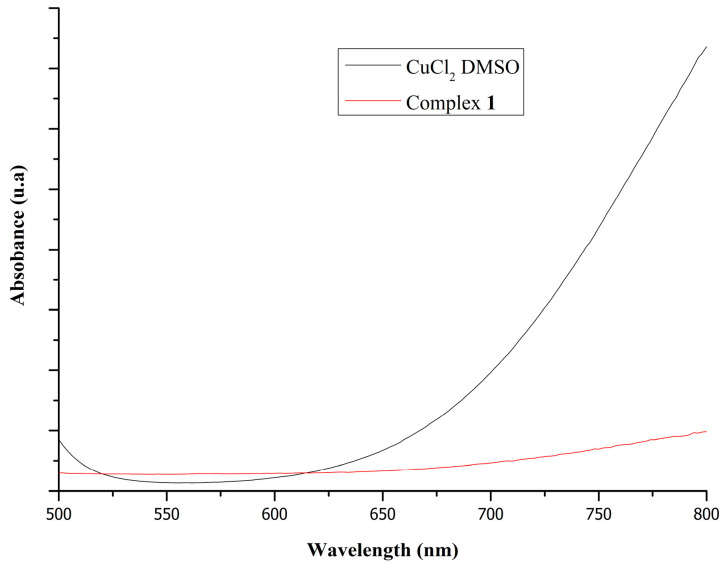
Comparison of the d–d transition observed for complex 1 (red) and the metal salt (black) at a concentration of 1 × 10^−3^ M, within the 500–800 nm range.

Metal complexes that allow *d–d* electronic transitions, generally exhibit weak and broad, bands in the visible region of the electromagnetic spectrum (λ > 380 nm) [48,49]. Considering the d^9^ electronic configuration of Cu^II^ complexes, the *d–d* transition (assigned as ν_1_ ^2^T_2_ → ^2^E_2_ (D)) could be expected; however, this transition is not fully observed in dilute solution (1 × 10^−5^ M) (Figure 3). However, the *d–d* electronic transitions could be observed at higher concentration (1 × 10^−3^ M) as can be seen in Figure 5 below.

It can be seen from Figure 5, that a discrete and discernible *d–d* electronic transition (red line) could be detected for the complex **1**, in DMSO solution. In comparison with copper chloride (black line) this electronic transition is less intense in accordance with the square planar geometry presented by the Cu^II^ complexes. The complex **2** presented a similar behavior in DMSO solution Figure S21.

**Figure 6:**
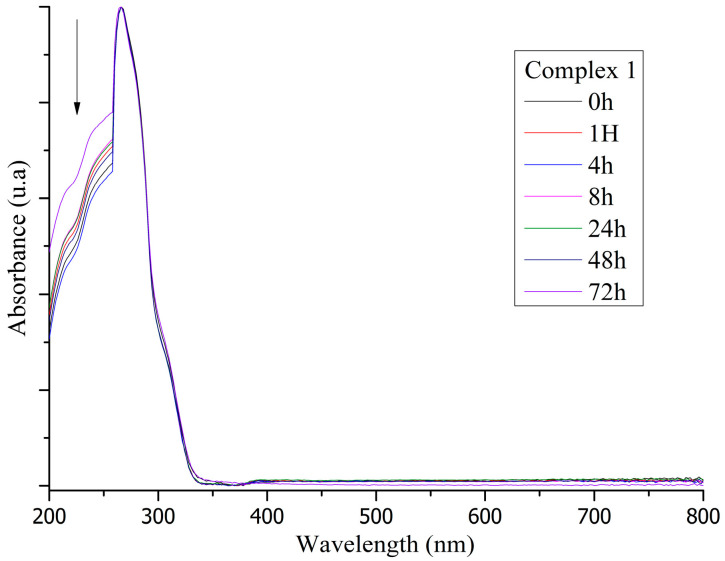
Time-dependent stability study of the complex 1 in DMSO at 1 × 10^−5^ M at 35 °C.

Given the importance of the structural stability of the complexes when in DMSO solution for biological investigations, a comprehensive stability assessment was performed by using a dilute DMSO solution (1 × 10^−5^ M), with the complexes maintained in solution for 48 h. In the monitored period, the complexes have demonstrated high stability in solution, and we did not detect structural disruption or solvolysis effect in a DMSO coordinating medium. Figure 6 shows the solution behavior monitored over 48 h for the complex **1**.

It can be seen from Figure 6, that the absorption profile did not show hypsochromic nor bathochromic shifts during 48 h of experiment. This behavior, along with the presence of distinct and noticeable *d–d* electronic transition (Figure 5) solution, it is a good proof that the complexes **1** and **2** are stable in DMSO solution, which is a necessary requirement for biological studies. Complex **2** presented a similar time-dependent stability in DMSO solution Figure S22.

### 3.4. Antileishmanial Investigation

The therapeutic approach for leishmaniasis is limited and comprises a high level of adverse effects [50,51]. The treatment predominantly relies on the use of pentavalent antimonials, which exhibit toxicity. In the Brazilian context, the treatment regimen includes mainly the administration of *N*-methylglucamine antimoniate, along with liposomal amphotericin B and pentamidine as second choice drugs [52]. However, the adverse effects associated with these pharmacological agents significantly offset their therapeutic efficacy, highlighting the need to explore alternative strategies for improved therapies and the development of new drugs [53,54].

**Figure 7:**
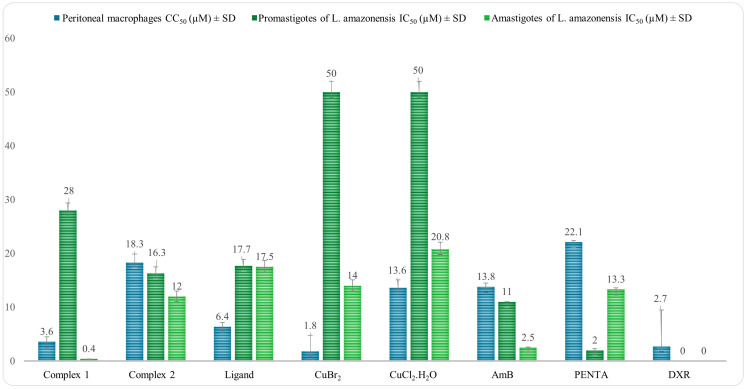
Comparative analysis of the biological activity, cytotoxic properties, and selectivity indices of novel complexes, unbound ligands, transition metal, and established pharmacological agents. AmB: amphotericin B, PENTA: pentamidine, DXR: doxorubicin.

Aiming to identify new antileishmanial agents and considering the biological diversity of the triazole derivatives and their coordination with metals, we evaluated the activity of the free ligand, two synthesized complexes, copper (II) chloride and bromide salts, and compared them with reference drugs (amphotericin B and pentamidine) against the *L. amazonensis* strain, related to cutaneous leishmaniases cases in Brazil (Figure 7 and Table 2).

The cytotoxic effects were evaluated against peritoneal macrophages. The two complexes synthesized exhibited different levels of cytotoxicity. **Complex 1** (CC_50_ = 3.6 µM) showed higher cytotoxicity than **complex 2** (CC_50_ = 18.3 µM), and a similar level of toxicity compared to DXR (CC_50_ = 2.7 µM). In contrast, the free ligand and the metal salts exhibited cytotoxicity ranging from moderate to high. Complex 2 demonstrated less cytotoxicity on macrophages than AmB (CC_50_ = 13.8 µM).

These results indicated that the free ligand and the complexes exhibited activity against both stages of the *Leishmania* (*L.*) *amazonensis* parasites. **Complex 2** (IC_50_ = 16.3 µM) was less active than the reference drugs tested against promastigote forms of *L. amazonensis*, AmB and PENTA (IC_50_ = 11.0 µM and 2.0 µM, respectively). Furthermore, **complex 2** was almost 2-fold more active than **complex 1** and demonstrated similar activity to the free ligand. Regarding the assay against intracellular amastigotes, **complex 2** (IC_50_ = 12.0 µM) exhibited similar antileishmanial activity to pentamidine (IC_50_ = 16.3 µM).

Interestingly, **complex 1** showed the highest selectivity index (SI = 9.7) among the compounds tested, demonstrating greater selectivity compared to amphotericin B and pentamidine. The promastigote assay revealed that **complex 1** (IC_50_ = 28.0 µM) was less active than the reference drugs (IC_50 AmB_ = 11.0 µM, IC_50 PENTA_ = 2.0 µM), **complex 2** (IC_50_ = 16.3 µM) and the free ligand (IC_50_ = 17.7 µM).

**Complex 1** demonstrated impressive potency against intracellular amastigote forms of *L. amazonensis*. Notably, **complex 1**, compared to the other compounds, tested in this work, was 6-fold and 33-fold more active than AmB and PENTA, respectively. Moreover, **complex 1** was 30-fold and 43-fold more active than **complex 2** and the free ligand, respectively.

The antileishmanial activity of triazole-analogues has been previously described in the literature. Almeida-Souza et al. (2020) demonstrated a hit triazole compound (IC_50_ = 17.8 µM) [50]. In contrast, using a comparative analysis with the compounds investigated in our work, **complex 1** exhibited 40-fold higher potency than the triazole compound.

In another study published by Yagmurlu et al. (2024) [55], copper complexes were tested against a different *Leishmania* strain (*L. major*), with all five complexes showing activity against both promastigote and amastigote stages. When comparing the activity against the amastigote stage, the best-performing compound reported by Yagmurlu et al. exhibited an IC_50_ of 0.39 µM and a selectivity index (SI) of approximately 10. These values are comparable to those obtained in the present study for *L. amazonensis*, where complex 1 displayed an IC_50_ of 0.4 µM and an SI of 9.7.

The Japanese initiative, Global Health Innovative Technology, in collaboration with Drugs for Neglected Diseases [56], established that selectivity is a critical parameter in the development of new drugs against infectious diseases. New compounds should exhibit a selectivity at least 10-fold higher than their cytotoxicity against mammalian cells, so compounds meeting this criterion can be considered “lead” compounds. Accordingly, the **complex 1** developed in this work meets this parameter and can be regarded as a successful lead compound for further in vivo studies against cutaneous leishmaniasis.

## 4. Conclusions

This study reports the design and synthesis of disubstituted 1,2,3-triazole ligands coordinated with Cu(II) metal centers. Two new Cu(II) complexes containing 1,4-disubstituted-1,2,3-triazole ligands were synthesized in good yields (57 and 60% for 1 and 2, respectively) and fully characterized. Single-crystal X-ray diffraction revealed a square-planar coordination geometry, with two ligand molecules coordinated to the Cu(II) center. Both complexes (1 and 2) exhibited structural stability in DMSO stability in solution and were subsequently evaluated in vitro for their biological activity. The cytotoxic effects of the complexes were assessed against murine peritoneal macrophages, and their antileishmanial activity was tested in vitro against both promastigote and intracellular amastigote forms of *Leishmania* (*L.*) *amazonensis*. Complex 1 was particularly effective against intracellular amastigotes, displaying a higher selectivity index compared to the reference drugs. Remarkably, complex 1 showed 6-fold and 33-fold greater activity than amphotericin B (AmB) and pentamidine (PENTA), respectively, against intracellular amastigotes. Moreover, complex 1 was 30-fold and 43-fold more potent as an antiamastigote agent compared to complex 2 and the free ligand, respectively. Overall, these results highlight complex 1 as a promising candidate for further development as a novel antileishmanial agent.

## Figures and Tables

**Table 1:** Main bands and attributions for the prepared compounds.

Compound	Wavelength (cm^−1^)		
	L1	Complex 1	Complex 2
ν(C-H)_ring_	3141	3132–3110	3128–3109
ν(C-H)_SP_^2^	2989	3051–3004	2999
ν(O-CH_3_)_sp_^3^	2941–2831	2941–2831	2933–2831
ν(C=N)	1562	1581	1579
ν(C=C)	1598	1614	1612
ν(C-O-CH_3_)	1230	1247	1249
ν(N-N)	1510	1514	1512

**Table 2:** Antileishmanial activity, cytotoxicity, and selectivity indexes of novel complexes of nitrogen-heterocyclic ligands and transition metals.

Compounds	Peritoneal Macrophages CC_50_^a^ (µM) ± SD	Promastigotes of *L. amazonensis* IC_50_^b^ (µM) ± SD	SI ^c^	Amastigotes of *L. amazonensis* IC_50_^d^ (µM) ± SD	SI ^e^
**Complex 1**	3.6 ± 0.9	28.0 ± 1.4	0.1	0.4± 0.0	9.7
**Complex 2**	18.3 ± 1.6	16.3 ± 1.2	1.1	12.0 ± 1.0	1.5
**Ligand**	6.4 ± 0.8	17.7 ± 1.2	0.4	17.5 ± 1.2	0.4
**CuBr_2_**	1.8 ± 3.0	>50.0 ± 2.0	>0.03	14.0 ±1.1	0.1
**CuCl_2_ 2H_2_O**	13.6 ± 1.5	>50.0 ± 2.0	>0.3	20.8 ± 1.3	0.6
**AmB ^f^**	13.8 ± 0.7	11.0 ± 0.0	1.2	2.5 ± 0.1	5.5
**PENTA ^g^**	22.1 ± 0.3	2.0 ± 0.3	11.0	13.3 ± 0.3	1.6
**DXR ^h^**	2.7 ± 6.8	Nd	Nd	Nd	Nd

The antileishmanial activity of complexes was evaluated against promastigote and amastigote forms of *Leishmania L. amazonensis*. Results are shown as mean ± standard deviation. Abbreviations: CC_50_ = half-maximal cytotoxic concentration; IC_50_ = half-maximal inhibitory concentration; SI = selectivity index; SD = standard deviation. ^(a)^: CC_50_ on murine peritoneal macrophages; ^(b)^: IC_50_ on promastigote forms of *Leishmania* (*L.*) *amazonensis*; ^(c)^: SI, IC_50_ on mammal cells/IC_50_ on promastigotes; ^(d)^: IC_50_ on amastigote forms of *Leishmania* (*L.*) *amazonensis*; ^(e)^: SI, IC_50_ on mammal cells/IC_50_ on amastigotes; ^(f, g)^: Drug reference treatment for *L. amazonensis*, PENTA = pentamidine, AmB = Amphotericin B; ^(h)^: positive control, doxorubicin (DXR) for peritoneal macrophages. Nd = means non-determined results.

## Data Availability

The original contributions presented in this study are included in the article and Supplementary Materials. The datasets used and/or analyzed during the current study are available from the corresponding author upon request.

## Supplementary Materials

The following supporting information can be downloaded at: https://www.mdpi.com/article/10.3390/pharmaceutics18010064/s1, Tables S1–S7, Figures S1–S24. Figure S1. Single crystals obtained after slow evaporation of the **Complex 1**; Figure S2. Single crystals obtained after slow evaporation of the **Complex 2**; Figure S3. Crystal structure of **Complex 2**. Hydrogen atoms are omitted for clarity. The symmetry operation used to generate equivalent atoms is #1: -x+1, -y, -z+1; Figure S4. Symmetry operators contained in the triclinic unit cell associated with the space group P$\bar{1}$ (left) and projection of the triclinic unit cell content of **Complex 1** along the same direction. For clarity, hydrogen atoms and solvent molecules have been omitted; Figure S5. Symmetry operators contained in the triclinic unit cell associated with the space group P$\bar{1}$ (left) and projection of the triclinic unit cell content of **Complex 2** along the same direction. For clarity, hydrogen atoms and solvent molecules have been omitted; Figure S6. DIAMOND projection of **Complex 1** illustrating intermolecular interactions via hydrogen bonds. Symmetry operations used to generate equivalent atoms: (#1) -x, -y+2, -z+1; (#2) -x+1, -y+1, -z+1; (#3) x, y-1, z; Figure S7. DIAMOND projection of Complex 2 illustrating intermolecular interactions via hydrogen bonds. Symmetry operations used to generate equivalent atoms: (#1) -x+1, -y, -z+1; (#2) x, y+1, z; Figure S8. Infrared (IR) vibrational spectrum of the ligand; Figure S9. Infrared (IR) vibrational spectrum of the **Complex 1** [Cu^II^Cl_2_(triazole)_2_]; Figure S10. Infrared (IR) vibrational spectrum of the **Complex 2** [Cu^II^Br_2_(triazole)_2_]; Figure S11. Full experimental mass spectrum ESI(+)-MS do **Complex 1**; Figure S12. Full experimental mass spectrum ESI(+)-MS do **Complex 2**; Figure S13. Experimental mass spectrum highlighting the exact mass and isotopic pattern of copper complexes, corresponding to the fragments of **Complex 1** with partial halogen loss at *m/z* 780.1753; Figure S14. Experimental mass spectra highlighting the exact mass and isotopic pattern of copper complex, corresponding to the fragments of **Complex 1** with complete halogen loss at *m/z* 745.2072; Figure S15. Experimental mass spectrum highlighting the exact mass and isotopic pattern of the protonated ligand fragment at *m/z* 342.1447; Figure S16. Experimental mass spectrum highlighting the exact mass and isotopic pattern of the copper (II) fragment from **Complex 1** with total halide loss, considering four ligands, at *m/z* 713.7386; Figure S17. Experimental mass spectrum showing the exact mass and isotopic distribution of the copper (II) fragment from **Complex 1** after total halide loss, considering four ligands, at *m/z* 713.7386; Figure S18. Experimental mass spectrum showing the exact mass and isotopic distribution of the copper (II) fragment from **Complex 2** after total halide loss, considering two ligands, at *m/z* 745.2055; Figure S19. Experimental mass spectrum highlighting the exact mass and isotopic pattern of the copper (II) fragment of **Complex 2** following complete halide loss, considering two ligands, at *m/z* 745.2055; Figure S20. Theoretical (red) and experimental (black) molecular electronic absorption. The **Complex 1** was diluted at 1.0 × 10^−5^ M in dichloromethane at 25 °C; Figure S21. Comparison of the d–d transition observed for **Complex 2** (red) and the metal salt (black) at a concentration of 1 × 10^−3^ M, within the 500–800 nm range; Figure S22. Time-dependent stability study of **Complex 2** in DMSO at a concentration of 1 × 10^−5^ M at 35°C; Figure S23. ^1^H NMR spectrum of Ligand in DMSO-d_6_ solution (δ in ppm); Figure S24. ^13^C NMR spectrum of Ligand in DMSO-d_6_ solution (δ in ppm). Table S1. Crystal data and structure refinement for the **Complexes 1** and **2**; Table S2. Table of bond lengths (Å) and bond angles (°) for **Complex 1**. The symmetry operation used to generate equivalent atoms is #1: -x, -y+2, -z+1; Table S3. Table of bond lengths (Å) and bond angles (°) for Complex 2. The symmetry operation used to generate equivalent atoms is #1 -x+1,-y,-z+1; Table S4. Bond lengths (Å) and angles (°) for **Complexes 1** and **2**; Table S5. Data on the major isotopic fragmentation patterns of the complexes, including their corresponding experimental *m/z*, calculated *m/z*, and mass errors; Table S6. Calculated energy levels, oscillator strength (*f*), and orbital transition analysis for selected lower-lying transitions; Table S7. IC_50_ values reported in the literature and for the studied complexes. Additional data from the structure can be obtained from the CCDC, CIF file for **Complex 1** CCDC no. 2483518 and CIF file for **Complex 2** CCDC no. 2483519.
