# Supplementary material for: New Copper (II) Complexes Based on 1,4-Disubstituted-1,2,3-Triazole Ligands with Promising Antileishmanial Activity
Source: Pharmaceutics. 2026 Jan 4;18(1):64. doi: 10.3390/pharmaceutics18010064 (PMC12845229; doi:10.3390/pharmaceutics18010064)
Supplement: Supplementary file 1 [file pharmaceutics-18-00064-s001.zip › pharmaceutics-3875923-supplementary.pdf]

1. Material and Methods

1.1. Melting Point

The melting point (m.p.) values were determined using a DF-3600 Instrutherm apparatus in a dry setup, with a working range of 50–300 °C and a capacity of up to three capillary tubes. Measurements were carried out at the Molecular Synthesis and Characterization Laboratory (LSCM), Institute of Chemistry, Federal University of Mato Grosso do Sul (UFMS), Campo Grande, MS, Brazil.

1.2. Elemental CHN Analysis

The carbon, hydrogen, and nitrogen content were determined at the Institute of Chemistry, University of São Paulo, using a VARIO EL elemental analyzer (Elementar Analysensysteme GmbH) on crystalline samples of each compound.

1.3. X-ray Diffractometry

Single crystals obtained from the crystallization of the complexes were analyzed at the Federal University of Santa Maria. X-ray diffraction data were collected using a SMART 1000 CCD Bruker diffractometer, a three-circle automatic instrument equipped with an area detector and operated with a graphite monochromator. Mo Kα radiation (λ = 0.71073 Å) was employed. Multi-scan methods were applied for absorption correction. The crystal structures were solved using SHELXS36[1] via direct methods, and all non-hydrogen atoms were refined anisotropically. Graphical representations of the crystal structures were generated using DIAMOND[2] and ORTEP[3] software.

1.4. Fourier Transform Infrared (FTIR) Spectroscopy in the Mid-Infrared Region

Spectral data in the infrared (IR) region were acquired using a BOMEM Hartmann & Braun spectrometer, The Michelson series MB-100, over the spectral range of 4000–500 cm^{-1}, with a resolution of 4 cm^{-1}, accumulation of 6 scans, and a scan rate of 0.2 cm/s. Samples were prepared by dispersing the compounds in spectroscopic-grade KBr and pressing them (~6 tons) using a mechanical press. All measurements were conducted at the Laboratory of Synthesis and Molecular Characterization (LSCM), Institute of Chemistry, UFMS, Campo Grande, MS, Brazil.

1.5. Molecular Absorption Spectroscopy in the Ultraviolet-Visible (UV-Vis) Region

Electronic absorption spectra in the ultraviolet and visible (UV-Vis) regions were recorded using a Lambda 60S spectrophotometer (PerkinElmer). Analyses were performed using solutions of the copper compounds in dimethyl sulfoxide at concentrations of approximately 1.0×10^{-5} mol L^{-1}, in quartz cells with a 3.0 mL capacity and 1 cm path length. All measurements were carried out at the Laboratory of Synthesis and Molecular Characterization (LSCM), Institute of Chemistry, UFMS, Campo Grande, MS, Brazil.

1.6. Density functional theory (DFT) and time-dependent density functional theory (TD-DFT) calculationsc

The theoretical calculations used to interpret the results were performed in partnership with Prof. Dr. Leandro M. C. Pinto from the Federal University of Mato Grosso do Sul, using Density Functional Theory (DFT) and Time-Dependent Density Functional Theory (TDDFT) as methodology. The density functional theory (DFT) calculations were performed using Becke's three-parameter hybrid exchange function combined with the Lee-Yang-Parr functional correlation (B3LYP) [4,5], SDD for Cu, and the 6-311G(d) basis set defined for C, N, O, Cl, and H using the Gaussian 16 software package[6]. The atomic coordinates, determined for the individual molecular structures by X-ray crystallographic analysis, were used for the DFT calculations. The vertical excitation energies were calculated using the time-dependent DFT (TD-DFT) methodology. The OM diagrams were reproduced using Avogadro (version 1.2.0)[7].

1.7. High-resolution mass spectrometry (HRMS) with electrospray ionization (ESI)

Mass spectrometry profiles were acquired using the Shimadzu LC-20AD UFLC Chromatograph, connected to the IES-Q-QTOF-MicroTOF III detector of the Mass Spectrometer (Bruker Daltonics). The instrument was operated in a positive ionization configuration (m/z 120-1200). The specimen was formulated at 100 µg/mL (acetonitrile/water 8:2) and introduced at a volume of 1 µL by manual infusion. An elution gradient was used, consisting of Water (phase A) and Acetonitrile (phase B), each supplemented with 0.1% formic acid. A 50% isocratic approach was applied over a 3-minute period. Quadrupole - Ion Energy was set at 5.0 eV. Collision Cell - Collision Energy was set at 10 eV. Data were analyzed using Bruker Data Analysis software version 4.2. Data were procured at the Natural Products and

Mass Spectrometry Laboratory — LAPNem, Federal University of Mato Grosso do Sul — UFMS, Campo Grande/MS.

1.8. Reagents for synthesis

All reagents and solvents of analytical grade were purchased from commercial suppliers and used without further purification.

	**Complex 2**	**Complex1**
**Empirical Formula**	$C_{36}H_{38}Br_2CuN_6O_8$	$C_{36}H_{38}Cl_2CuN_6O_8$
**Formula weight (g/mol)**	906,08	817.16
**Temperature T° (K)**	100(2)	100(2)
**Crystal system**	Triclinic	Triclínic
**Space group**	$P\bar{1}$	$P\bar{1}$
**Unit cell dimensions (Å)**	7.99382(3)	7.9792(3)
**b (Å)**	8.4400(3)	8.3034(3)
**c (Å)**	14.4055(5)	15.4355(6)
**α (º)**	97.386(2)	85.6810(10)
**β (º)**	93.469(2)	87.1280(10)
**ɣ (º)**	105.8820(10)	63.2920(10)
**Volume (Å^3)**	922.37(6)	910.82(6)
**Number of elementary formulas (Z)**	Z = 1	Z = 1
**Calculated density Mg/m^3**	1.631	1.490
**Absorption coefficient (mm^{-1})**	2.818	0.807
**F (000)**	459	423
**Crystal size (mm)**	0.23 x 0.22 x 0.19	0.33 x 0.28 x 0.28
**Theta range for data collection**	2.54 a 29.18º	2.65 a 29.17º
**Limiting indices**	-10<=h<=10<br>-11<=k<=11<br>-18<=l<=19	-10<=h<=10<br>-11<=k<=11<br>-21<=l<=21
**Reflections collected**	16238	24323
**Independent reflections**	4963 [R(int) = 0.0276]	4902 [R(int) = 0.0208]
**Absorption correction**	Semi-empirical from equivalents	Semi-empirical from equivalents
**Data / restraints / parameters**	4963 / 0 / 241	4902 / 2 / 241
**Final R indices [I> 2sigma(I)]**	R1 = 0.0277<br>wR2 = 0.0573	R1 = 0.0236<br>wR2 = 0.0663
**R indexices (all data)**	R1 = 0.0381<br>wR2 = 0.0602	R1 = 0.0249<br>wR2 = 0.0671
**Largest diff. peak and hole. (e/Å^3)**	0.448 e -0.394	0.461 e -0.390

Table S1. Crystal data and structure refinement for the **Complexes 1** and **2**.

Cu-N(1)	1.9970(9)
Cu-N(1)#1	1.9970(9)
Cu-Cl	2.2411(3)
Cu-Cl#1	2.2411(3)
O(1)-C(6)	1.3668(14)
O(1)-C(15)	1.4301(18)
O(2)-C(11)	1.3605(13)
O(2)-C(16)	1.4332(14)
O(4)-C(13)	1.3615(13)
O(4)-C(18)	1.4312(14)
O(3)-C(12)	1.3778(13)
O(3)-C(17)	1.4328(16)
N(1)-N(2)	1.3220(12)
N(1)-C(2)	1.3658(13)
N(2)-N(3)	1.3419(12)
N(3)-C(1)	1.3545(14)
N(3)-C(9)	1.4353(13)
C(3)-C(8)	1.3937(15)
C(3)-C(4)	1.4053(15)
C(3)-C(2)	1.4674(14)
C(11)-C(10)	1.3960(14)
C(11)-C(12)	1.3984(15)
C(10)-C(9)	1.3855(14)
C(4)-C(5)	1.3807(16)
C(9)-C(14)	1.3852(15)
C(2)-C(1)	1.3798(14)
C(12)-C(13)	1.4018(15)
C(13)-C(14)	1.3954(14)
C(6)-C(7)	1.3921(17)
C(6)-C(5)	1.3964(17)
C(7)-C(8)	1.3988(15)

N(1)-Cu-N(1)#1	180.0
N(1)-Cu-Cl	88.65(3)
N(1)#1-Cu-Cl	91.35(3)
N(1)-Cu-Cl#1	91.35(3)
N(1)#1-Cu-Cl#1	88.65(3)
Cl-Cu-Cl#1	180.0
C(6)-O(1)-C(15)	117.27(11)
C(11)-O(2)-C(16)	115.60(9)
C(13)-O(4)-C(18)	117.15(9)
C(12)-O(3)-C(17)	114.99(10)
N(2)-N(1)-C(2)	110.76(9)
N(2)-N(1)-Cu	117.85(7)
C(2)-N(1)-Cu	130.89(7)
N(1)-N(2)-N(3)	105.81(8)
N(2)-N(3)-C(1)	111.64(9)
N(2)-N(3)-C(9)	120.69(9)
C(1)-N(3)-C(9)	127.50(9)
C(8)-C(3)-C(4)	118.81(10)
C(8)-C(3)-C(2)	122.89(10)
C(4)-C(3)-C(2)	118.29(10)
O(2)-C(11)-C(10)	123.30(10)
O(2)-C(11)-C(12)	115.94(10)
C(10)-C(11)-C(12)	120.75(10)
C(9)-C(10)-C(11)	118.10(10)
C(5)-C(4)-C(3)	120.62(11)
C(10)-C(9)-C(14)	122.93(10)
C(10)-C(9)-N(3)	117.57(9)
C(14)-C(9)-N(3)	119.46(9)
N(1)-C(2)-C(1)	106.61(9)
N(1)-C(2)-C(3)	125.13(9)
C(1)-C(2)-C(3)	128.18(10)
O(3)-C(12)-C(11)	121.25(10)
O(3)-C(12)-C(13)	119.23(10)
C(11)-C(12)-C(13)	119.37(10)
N(3)-C(1)-C(2)	105.17(9)
O(4)-C(13)-C(14)	123.92(10)
O(4)-C(13)-C(12)	115.52(9)
C(14)-C(13)-C(12)	120.56(10)
C(9)-C(14)-C(13)	118.25(10)
O(1)-C(6)-C(7)	124.89(11)
O(1)-C(6)-C(5)	115.22(11)
C(7)-C(6)-C(5)	119.89(11)
C(6)-C(7)-C(8)	119.67(11)
C(3)-C(8)-C(7)	120.73(11)
C(4)-C(5)-C(6)	120.26(11)

Table S2. Table of bond lengths (Å) and bond angles (°) for **Complex 1**. The symmetry operation used to generate equivalent atoms is #1: -x, -y+2, -z+1.

Br-Cu	2.4314(2)	N(1)-Cu-Br	90.26(4)
N(3)-N(2)	1.3406(19)	N(1)#1-Cu-Br	89.74(4)
N(3)-C(1)	1.350(2)	Br#1-Cu-Br	180.0
N(3)-C(9)	1.432(2)	C(12)-O(3)-C(17)	113.31(14)
O(4)-C(13)	1.361(2)	C(11)-O(2)-C(16)	116.48(13)
O(4)-C(18)	1.432(2)	C(6)-O(1)-C(15)	117.30(15)
Cu-N(1)	1.9503(14)	N(2)-N(1)-C(2)	111.09(14)
Cu-N(1)#1	1.9504(14)	N(2)-N(1)-Cu	116.86(11)
O(3)-C(12)	1.3761(19)	C(2)-N(1)-Cu	131.97(11)
O(3)-C(17)	1.437(2)	N(1)-N(2)-N(3)	105.51(13)
O(2)-C(11)	1.361(2)	N(1)-C(2)-C(1)	106.14(15)
O(2)-C(16)	1.430(2)	N(1)-C(2)-C(3)	124.92(15)
O(1)-C(6)	1.371(2)	C(1)-C(2)-C(3)	128.93(16)
O(1)-C(15)	1.432(2)	N(3)-C(1)-C(2)	105.69(15)
N(1)-N(2)	1.3227(19)	C(4)-C(3)-C(8)	118.45(16)
N(1)-C(2)	1.365(2)	C(4)-C(3)-C(2)	119.19(15)
C(2)-C(1)	1.375(2)	C(8)-C(3)-C(2)	122.31(16)
C(2)-C(3)	1.468(2)	C(7)-C(8)-C(3)	120.56(17)
C(3)-C(4)	1.393(2)	O(2)-C(11)-C(10)	124.04(16)
C(3)-C(8)	1.401(2)	O(2)-C(11)-C(12)	115.42(15)
C(8)-C(7)	1.380(2)	C(10)-C(11)-C(12)	120.54(16)
C(11)-C(10)	1.394(2)	C(4)-C(5)-C(6)	118.99(17)
C(11)-C(12)	1.398(3)	C(9)-C(14)-C(13)	118.01(16)
C(5)-C(4)	1.389(2)	C(5)-C(4)-C(3)	121.54(16)
C(5)-C(6)	1.393(3)	C(10)-C(9)-C(14)	123.14(15)
C(14)-C(9)	1.388(2)	C(10)-C(9)-N(3)	117.98(15)
C(14)-C(13)	1.393(2)	C(14)-C(9)-N(3)	118.88(15)
C(9)-C(10)	1.384(2)	C(8)-C(7)-C(6)	120.24(17)
C(7)-C(6)	1.392(3)	C(9)-C(10)-C(11)	117.99(16)
C(12)-C(13)	1.396(2)	O(1)-C(6)-C(7)	115.38(16)
		O(1)-C(6)-C(5)	124.39(17)
N(2)-N(3)-C(1)	111.56(13)	C(7)-C(6)-C(5)	120.21(16)
N(2)-N(3)-C(9)	120.18(14)	O(3)-C(12)-C(13)	120.02(16)
C(1)-N(3)-C(9)	128.26(15)	O(3)-C(12)-C(11)	120.16(16)
C(13)-O(4)-C(18)	117.45(14)	C(13)-C(12)-C(11)	119.75(15)
N(1)-Cu-N(1)#1	180.0	O(4)-C(13)-C(14)	124.38(16)
N(1)-Cu-Br#1	89.74(4)	O(4)-C(13)-C(12)	115.06(15)
N(1)#1-Cu-Br#1	90.26(4)	C(14)-C(13)-C(12)	120.55(16)

Table S3. Table of bond lengths (Å) and bond angles (°) for **Complex 2**. The symmetry operation used to generate equivalent atoms is #1 -x+1,-y,-z+1.

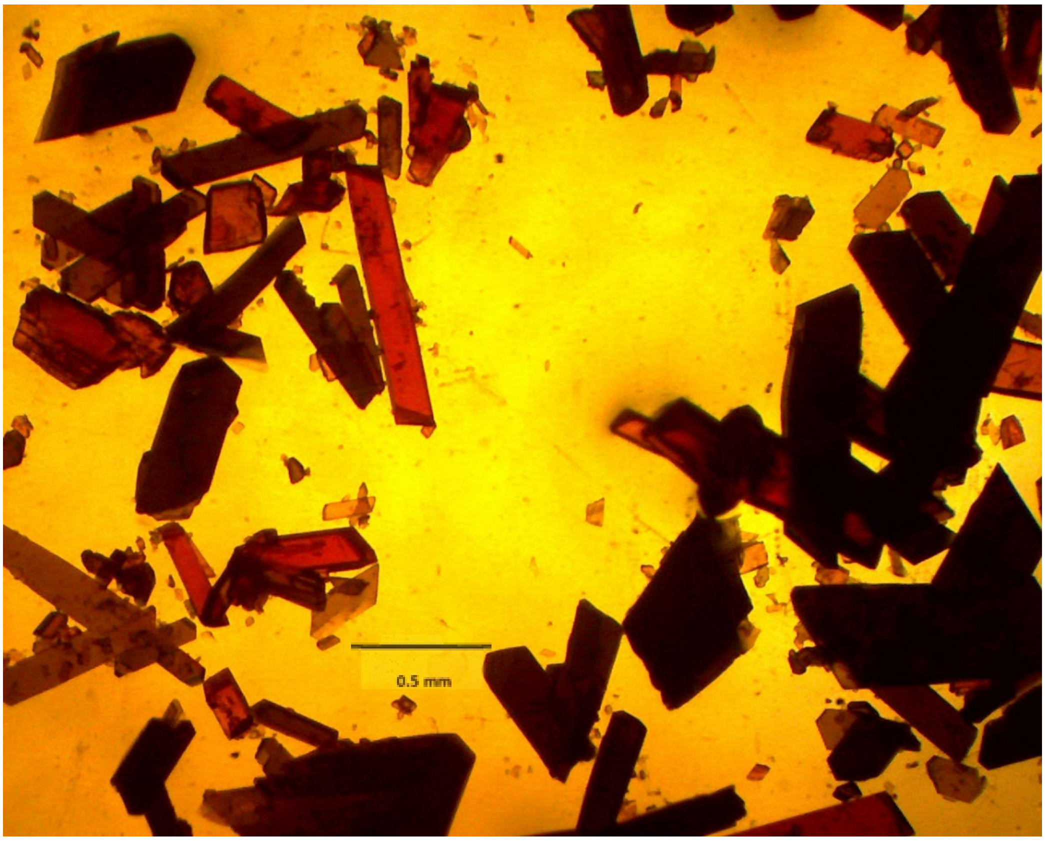

Figure S1: Single crystals obtained after slow evaporation of the **Complex 1**.

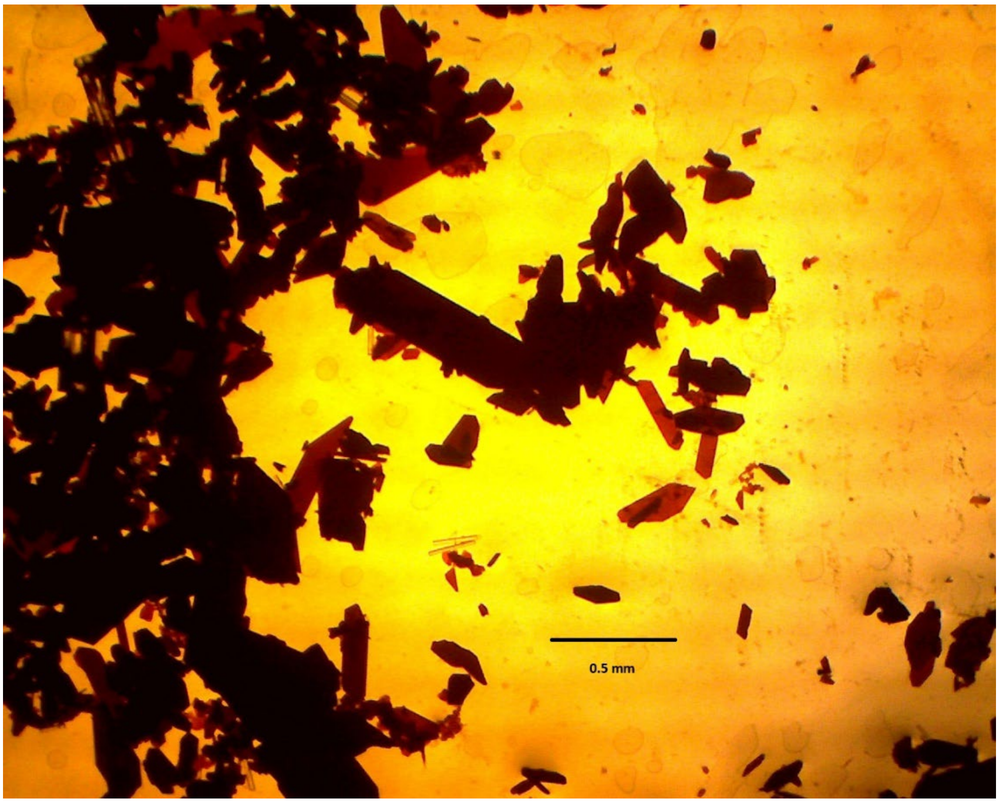

Figure S2: Single crystals obtained after slow evaporation of the **Complex 2**.

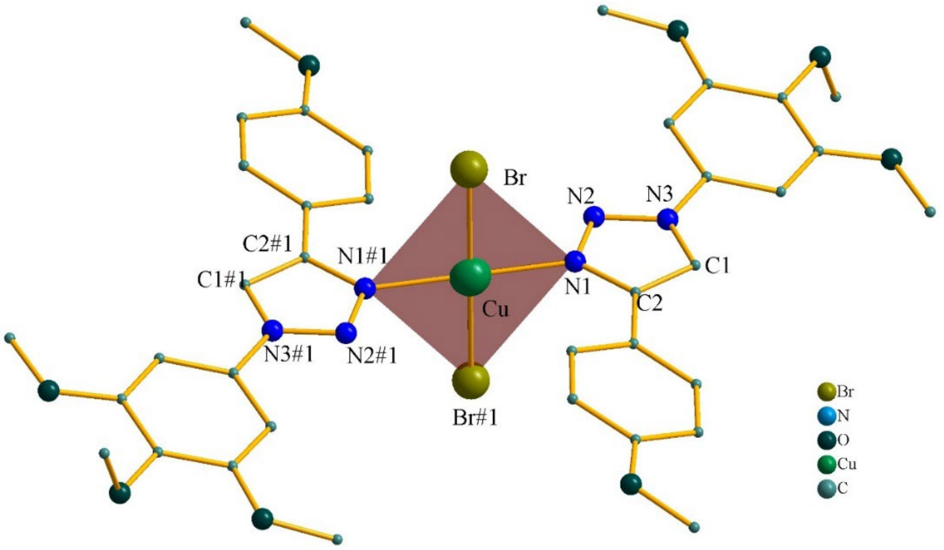

Figure S3: Crystal structure of **Complex 2**. Hydrogen atoms are omitted for clarity. The symmetry operation used to generate equivalent atoms is #1: -x+1, -y, -z+1.

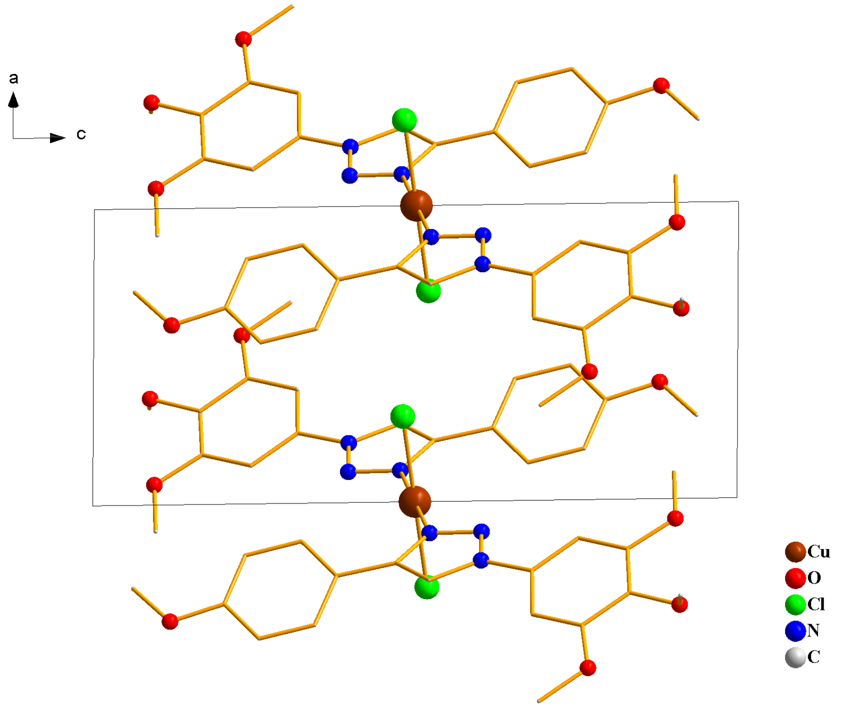

Figure S4: Symmetry operators contained in the triclinic unit cell associated with the space group P$\bar{1}$ (left) and projection of the triclinic unit cell content of **Complex 1** along the same direction. For clarity, hydrogen atoms and solvent molecules have been omitted.

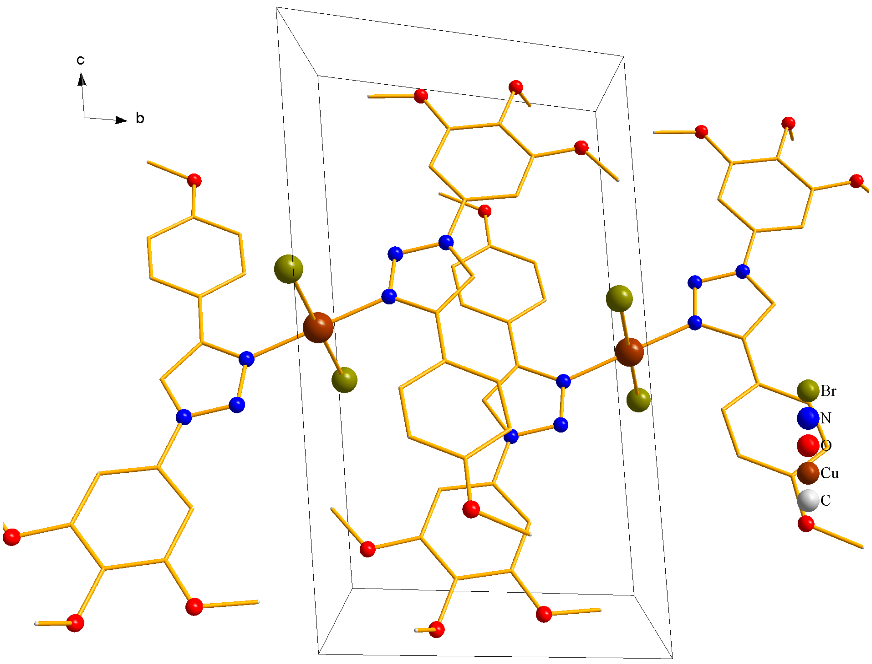

Figure S5: Symmetry operators contained in the triclinic unit cell associated with the space group P$\bar{1}$ (left) and projection of the triclinic unit cell content of **Complex 2** along the same direction. For clarity, hydrogen atoms and solvent molecules have been omitted.

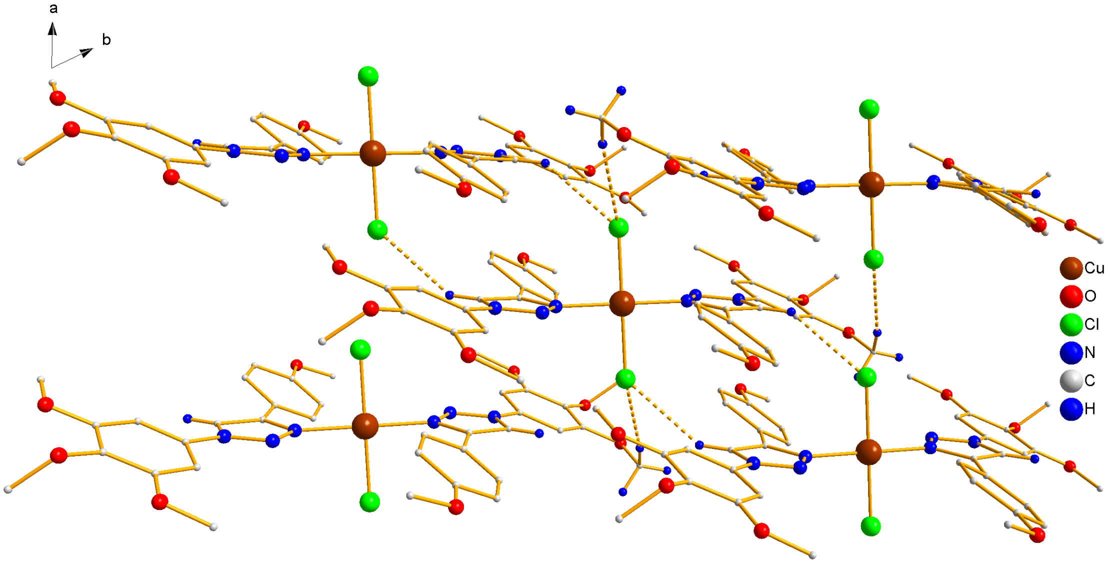

Figure S6: DIAMOND projection of **Complex 1** illustrating intermolecular interactions via hydrogen bonds. Symmetry operations used to generate equivalent atoms: (#1) -x, -y+2, -z+1; (#2) -x+1, -y+1, -z+1; (#3) x, y-1, z.

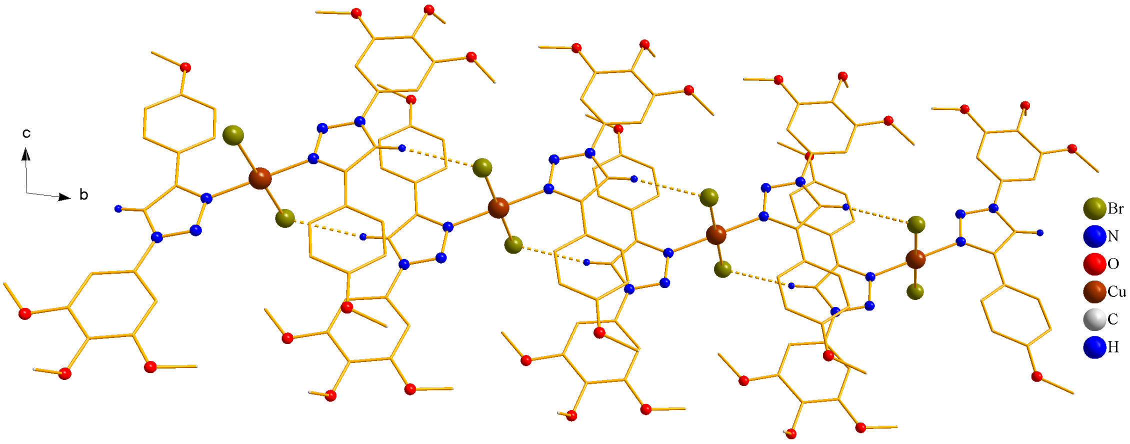

Figure S7: DIAMOND projection of Complex 2 illustrating intermolecular interactions via hydrogen bonds. Symmetry operations used to generate equivalent atoms: (#1) -x+1, -y, -z+1; (#2) x, y+1, z.

Complex	D-H...A	d(D-H)	d(H...A)	d(D...A)	<(DHA)
**1**	C(1)-H(1)...Cl#2	0.95	2.93	3.7550(11)	146.0
	C(16)-H(16B)...Cl#3	0.95	2.95	3.8865(13)	167.38
**2**	C(1)-H(1)...Br#2	0.95	2.85	3.7567(18)	160.4

Symmetry operators for **Complex 1**: #1 -x,-y+2,-z+1; #2 -x+1,-y+1,-z+1; #3 x,y-1,z
Symmetry operators for **Complex 2**: #1 -x+1,-y,-z+1; #2 x,y+1,z

Table S4. Bond lengths (Å) and angles (°) for **Complexes 1** and **2**.

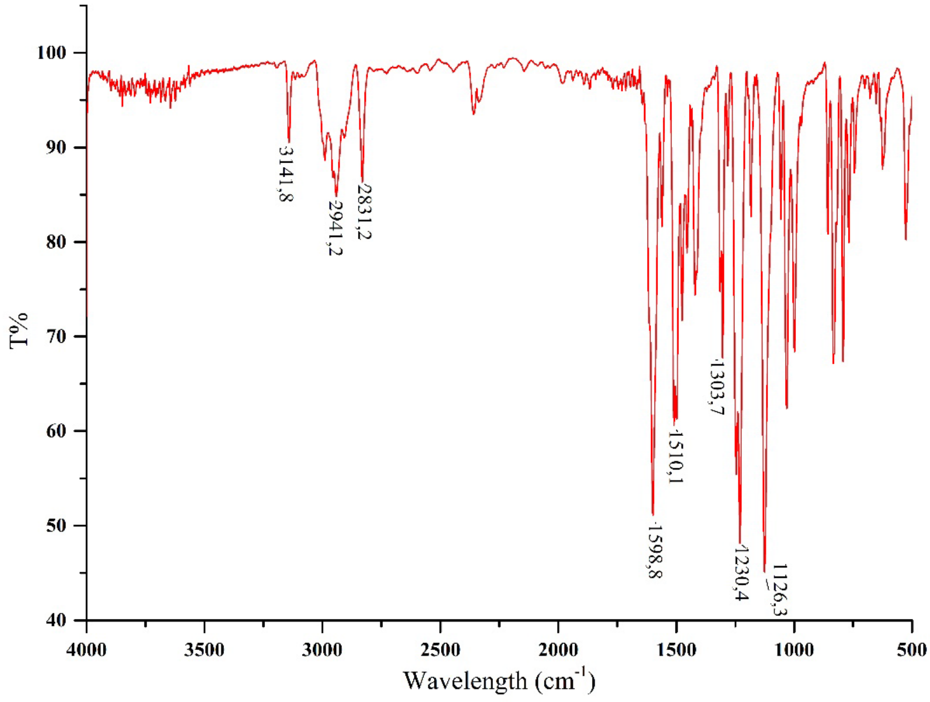

Figure S8: Infrared (IR) vibrational spectrum of the ligand.

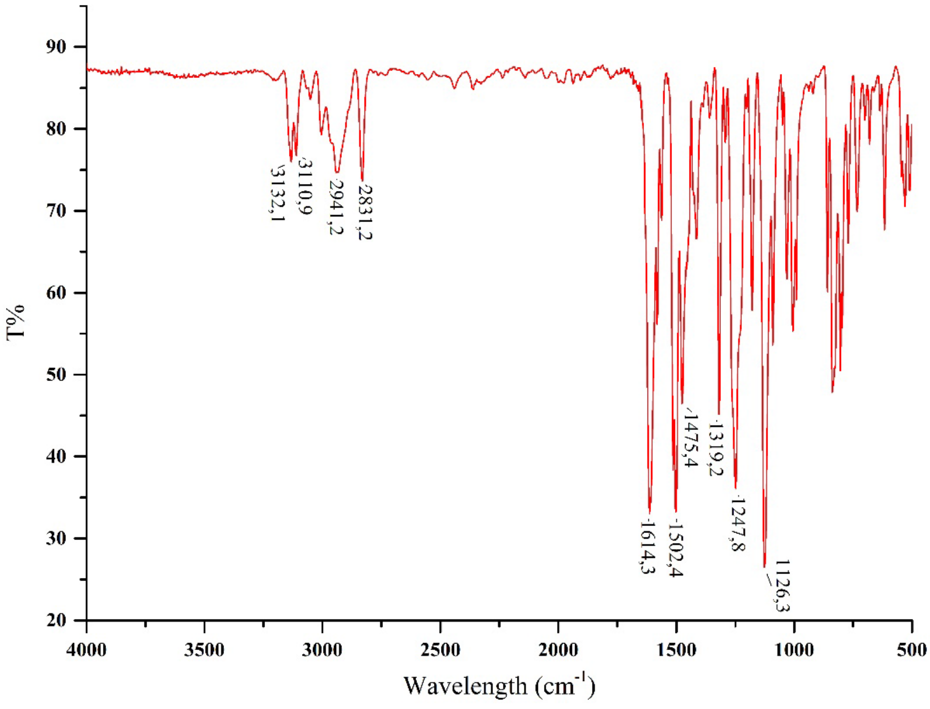

Figure S9: Infrared (IR) vibrational spectrum of the **Complex 1** [$Cu^{II}Cl_2$(triazole)$_2$].

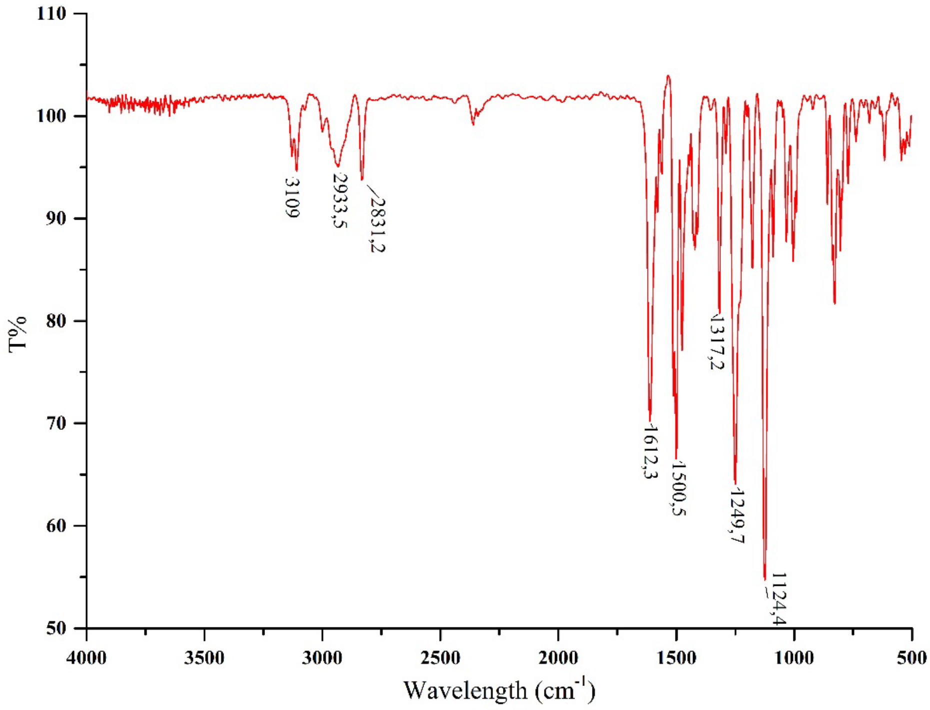

Figure S10: Infrared (IR) vibrational spectrum of the **Complex 2** [$Cu^{II}Br_2$(triazole)$_2$].

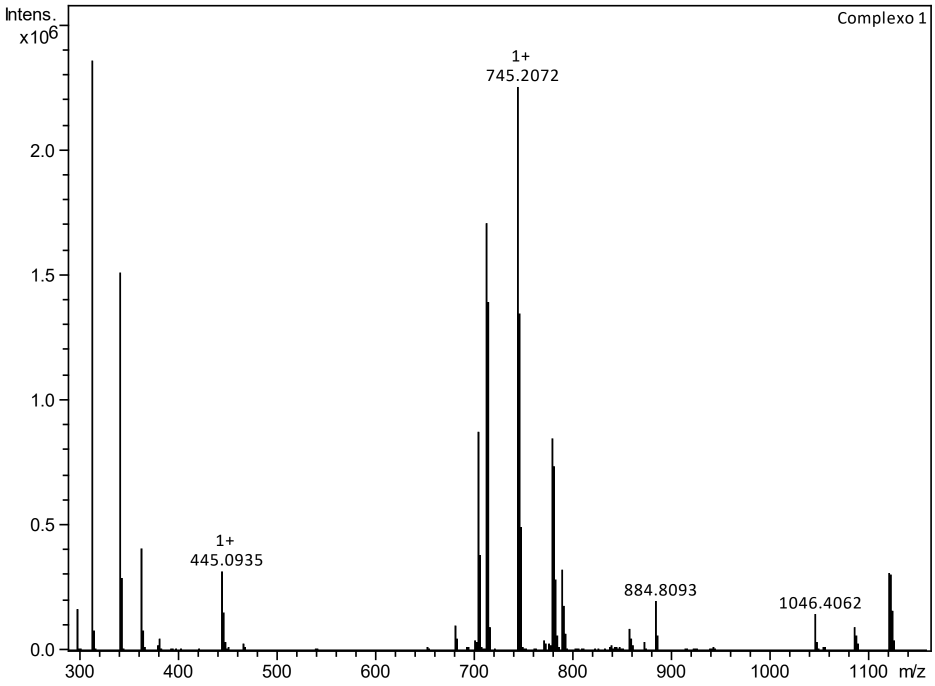

Figure S11: Full experimental mass spectrum ESI(+)-MS do **Complex 1**.

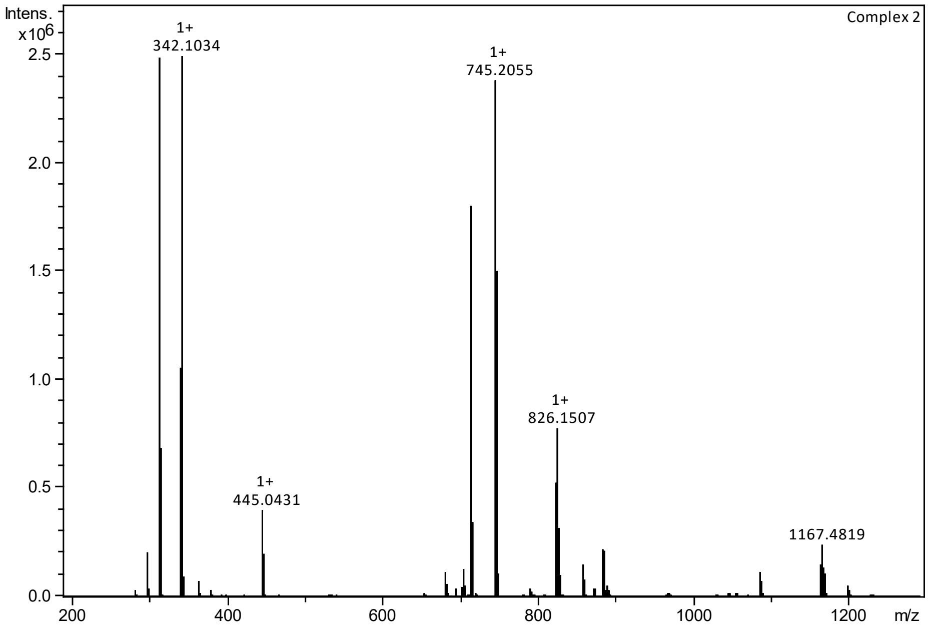

Figure S12: Full experimental mass spectrum ESI(+)-MS do **Complex 2**.

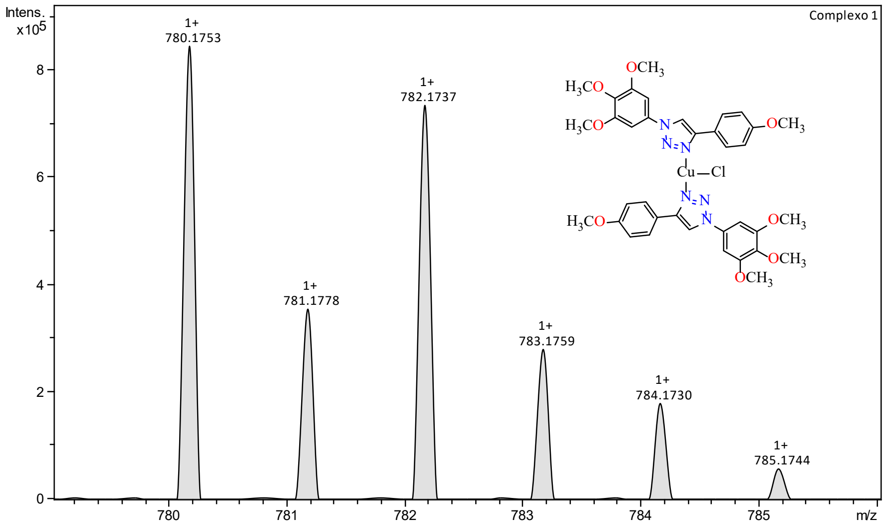

Figure S13: Experimental mass spectrum highlighting the exact mass and isotopic pattern of copper complexes, corresponding to the fragments of **Complex 1** with partial halogen loss at m/z 780.1753.

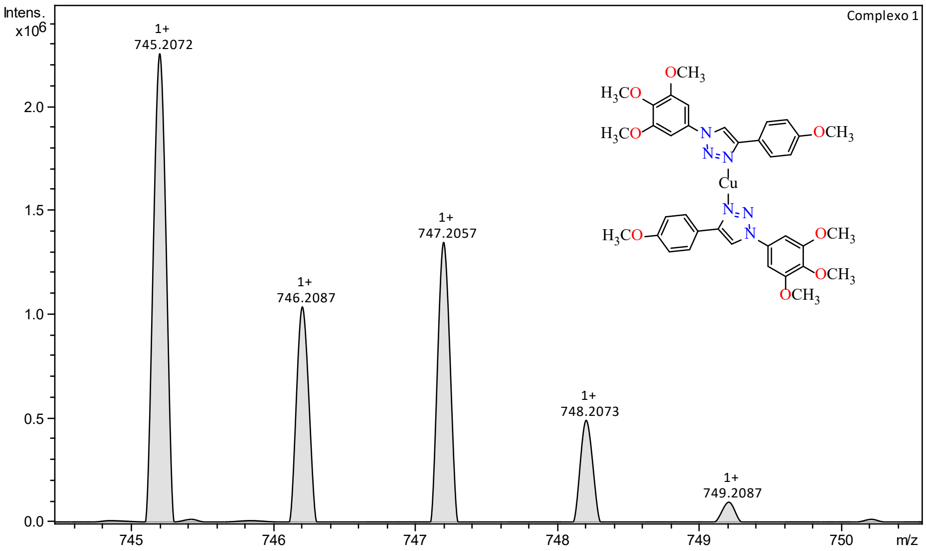

Figure S14: Experimental mass spectra highlighting the exact mass and isotopic pattern of copper complex, corresponding to the fragments of **Complex 1** with complete halogen loss at m/z 745.2072.

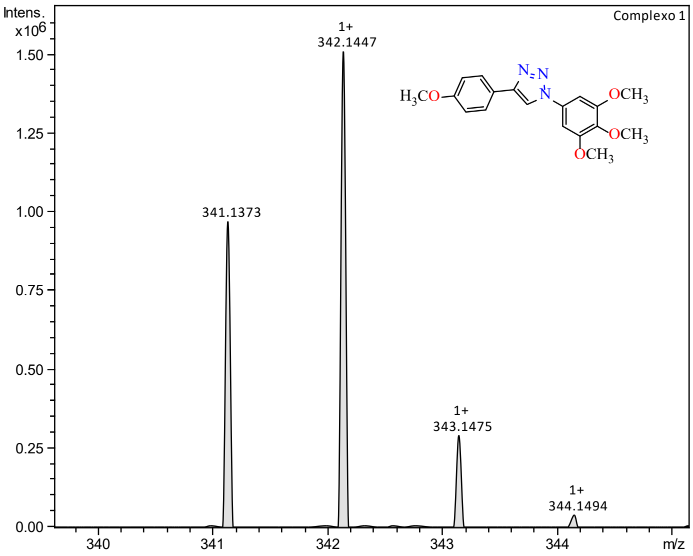

Figure S15: Experimental mass spectrum highlighting the exact mass and isotopic pattern of the protonated ligand fragment at m/z 342.1447.

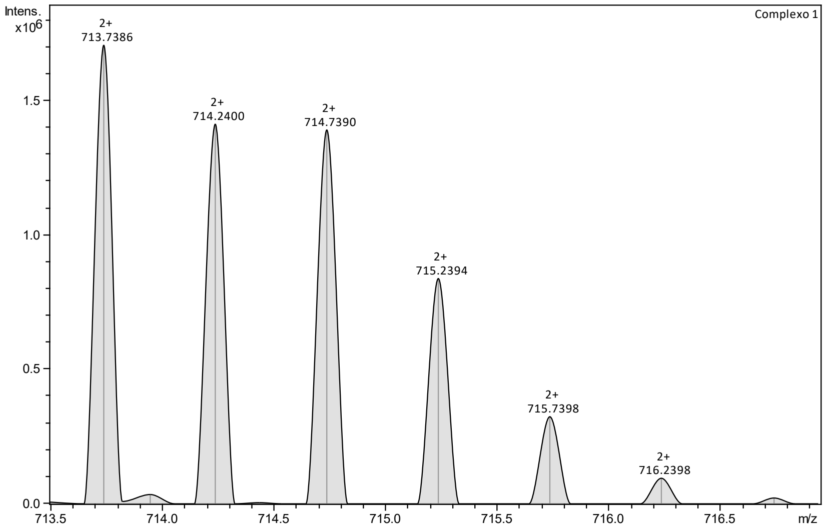

Figure S16: Experimental mass spectrum highlighting the exact mass and isotopic pattern of the copper (II) fragment from **Complex 1** with total halide loss, considering four ligands, at m/z 713.7386.

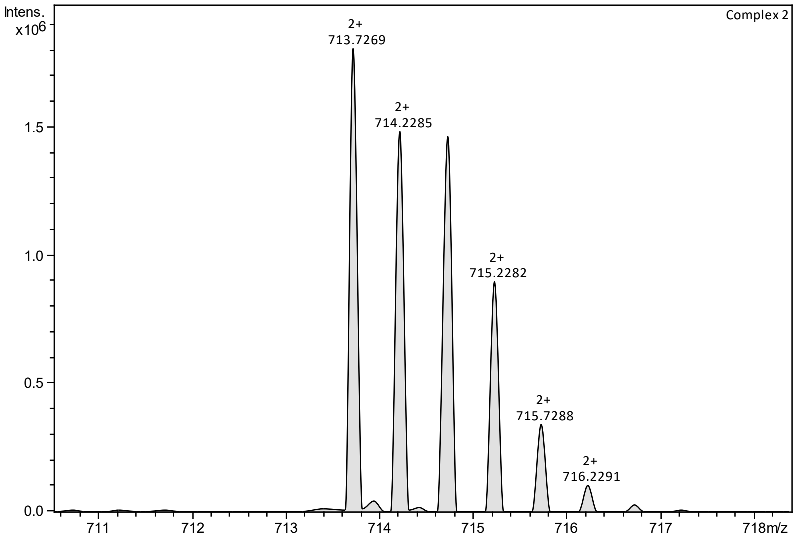

Figure S17: Experimental mass spectrum showing the exact mass and isotopic distribution of the copper (II) fragment from **Complex 1** after total halide loss, considering four ligands, at m/z 713.7386.

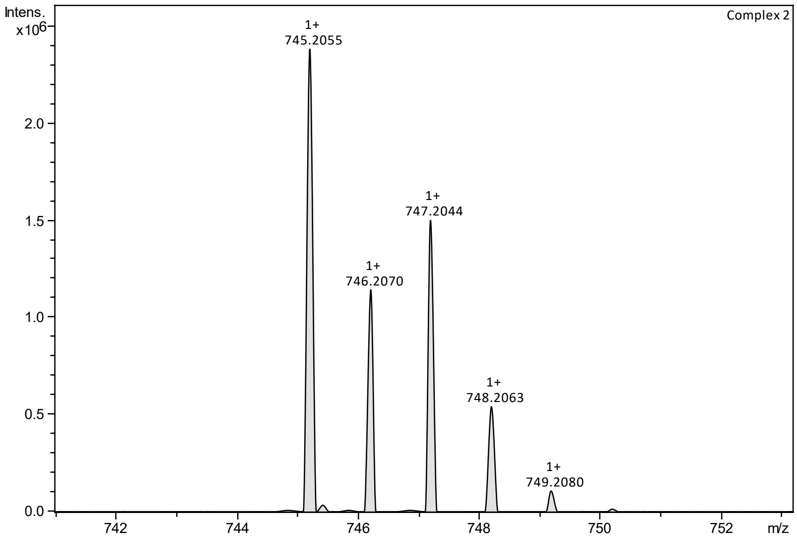

Figure S18: Experimental mass spectrum showing the exact mass and isotopic distribution of the copper (II) fragment from **Complex 2** after total halide loss, considering two ligands, at m/z 745.2055.

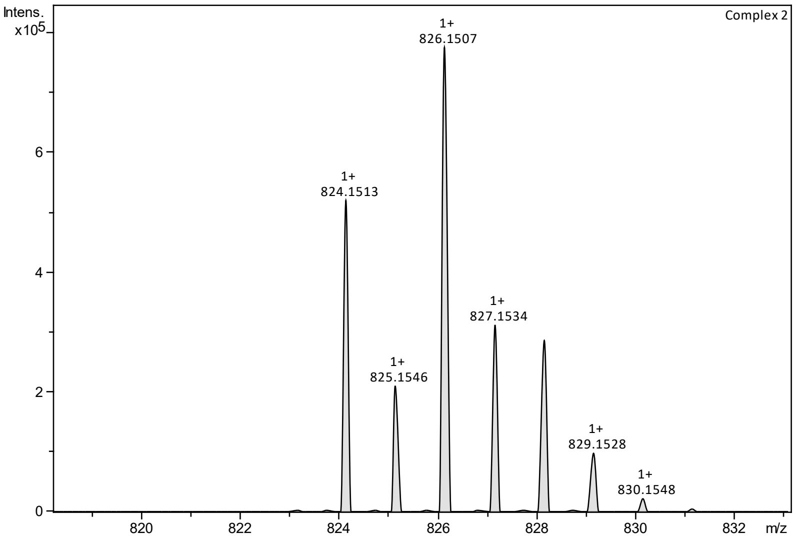

Figure S19: Experimental mass spectrum highlighting the exact mass and isotopic pattern of the copper (II) fragment of **Complex 2**following complete halide loss, considering two ligands, at m/z 745.2055.

**Complexes**	Formula	Molecular Mass	Fragment	*m/z* Experimental	*m/z* Calculate	Error (PPM)
**1**	$C_{36}H_{38}Cl_2CuN_6O_8$	817.18	$[C_{18}H_{20}N_3O_4]^+$	342.1447	342.1454	2.04591
			$[C_{72}H_{76}CuN_{12}O_{16}]^{+2}$	713.7386	713.7399	1.82139
			$[C_{36}H_{38}CuN_6O_8]^+$	745.2072	745.2047	3.35478
			$[C_{36}H_{18}ClCuN_6O_8]^+$	780.1753	780.1736	2.17900
			$[C_{54}H_{57}CuN_9O_{12}]^+$	1086.3467	1086.3423	4.05028
			$[C_{54}H_{57}ClCuN_9O_{12}]^+$	1121.3155	1121.3111	3.92397
**2**	$C_{36}H_{38}Br_2CuN_6O_8$	906.08	$[C_{18}H_{20}N_3O_4]^+$	342.1034	342.1376	>100
			$[C_{72}H_{76}CuN_{12}O_{16}]^{+2}$	713.7269	713.7399	18.4941
			$[C_{36}H_{18}CuN_6O_8]^+$	745.2055	745.2047	1.0735
			$[C_{36}H_{38}BrCuN_6O_8]^+$	824.1513	824.1230	33.97
			$[C_{54}H_{57}CuN_9O_{12}]^+$	1086.5101	1086.3423	>150
			$[C_{54}H_{57}BrCuN_9O_{12}]^+$	1165.4816	1165.2606	>180

Table S5. Data on the major isotopic fragmentation patterns of the complexes, including their corresponding experimental m/z, calculated m/z, and mass errors.

State	λ (nm)	*F*	largest contribution	lower contribution	description
S1	340.5	0.0272	H–5(β) → L(β) (49%)<br>H–10(β) → L(β) (17%)	H–3(β) → L(β) (10%);<br>H–28(β) → L(β) (8%);<br>H–6(β) → L(β) (7%);<br>H–7(β) → L(β) (2%)	LMCT
S2	320.0	0.0159	H–6(β) → L(β) (54%)	H–10(β) → L(β) (12%);<br>H–5(β) → L(β) (11%);<br>H–18(β) → L(β) (4%);<br>H–41(β) → L(β) (3%);<br>H–33(β) → L(β) (2%);<br>H–30(β) → L(β) (3%);<br>H–3(β) → L(β) (2%);<br>H–1(β) → L(β) (2%)	LMCT
S3	308.1	0.0563	H–10(β) → L(β) (52%)	H–28(β) → L(β) (14%);<br>H–6(β) → L(β) (14%);<br>H–2(α) → L(α) (2%);<br>H–30(β) → L(β) (2%);<br>H–25(β) → L(β) (2%);<br>H–9(β) → L(β) (2%);<br>H–3(β) → L(β) (2%)	LMCT
S4	265.7	0.0122	H–2(α) → L+1(α) (48%)<br>H(α) → L+1(α) (29%)	H–2(α) → L(α) (4%);<br>H(α) → L(α) (3%);<br>H–10(β) → L(β) (3%)	IL + XLCT
S5	255.0	0.0173	H–2(β) → L+1(β) (42%)<br>H(β) → L+1(β) (17%)	H–1(β) → L+1(β) (11%);<br>H–3(α) → L(α) (5%);<br>H–6(β) → L+1(β) (5%);<br>H–7(α) → L(α) (3%);	IL + XLCT

				H–4(α) → L(α) (2%)	
S6	249.5	0.0245	H–10(α) → L(α) (32%)<br>H–9(β) → L+1(β) (31%)	H–2(α) → L(α) (7%);<br>H(α) → L(α) (6%);<br>H(β) → L+1(β) (3%);<br>H–1(α) → L(α) (2%);<br>H–9(β) → L+9(β) (2%);<br>H–10(α) → L+1(α) (2%);<br>H–10(α) → L+8(α) (2%);<br>H–3(α) → L(α) (2%)	IL + XLCT
S7	245.1	0.0371	H–3(β) → L+1(β) (58%)	H–1(α) → L(α) (10%);<br>H–1(β) → L+1(β) (6%);<br>H–2(B) → L+1(B) (5%);<br>H–2(α) → L+1(α) (3%);<br>H–1(α) → L+1(α) (3%);<br>H(α) → L+1(α) (2%);<br>H–3(α) → L+1(α) (2%);<br>H–5(β) → L+1(β) (2%)	IL + XLCT
S8	241.6	0.0231	H–4(β) → L+1(β) (29%)	H–1(β) → L+2(β) (11%);<br>H–3(β) → L+2(β) (9%);<br>H(β) → L+2(β) (9%);<br>H–3(α) → L(α) (8%);<br>H–4(β) → L+2(β) (6%);<br>H–2(α) → L(α) (4%);<br>H–1(α) → L(α) (4%);<br>H–1(α) → L+1(α) (4%);<br>H(α) → L(α) (3%);<br>H–2(β) → L+2(β) (3%);<br>H–3(α) → L+1(α) (2%)	IL + XLCT
S9	238.4	0.0102	H–4(β) → L+2(β) (32%)<br>H–1(α) → L+1(α) (24%)	H(β) → L+2(β) (13%);<br>H(α) → L+1(α) (11%);<br>H–6(α) → L+1(α) (4%);<br>H–4(β) → L+1(β) (3%);<br>H–2(β) → L+2(β) (3%)	IL + XLCT

Table S6. Calculated energy levels, oscillator strength (*f*), and orbital transition analysis for selected lower-lying transitions.

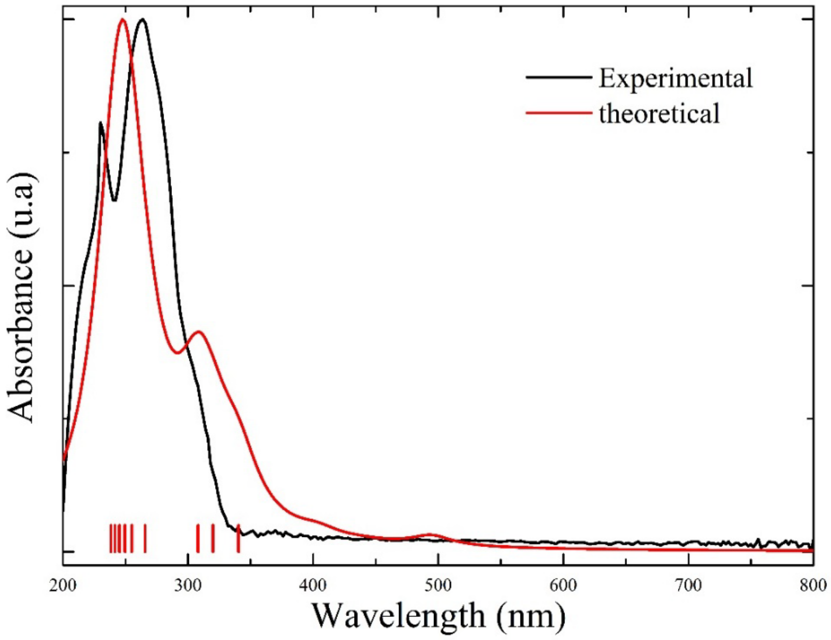

Figure S20: Theoretical (red) and experimental (black) molecular electronic absorption. The **Complex 1** was diluted at 1.0 X 10-5 M in dichloromethane at 25ºC.

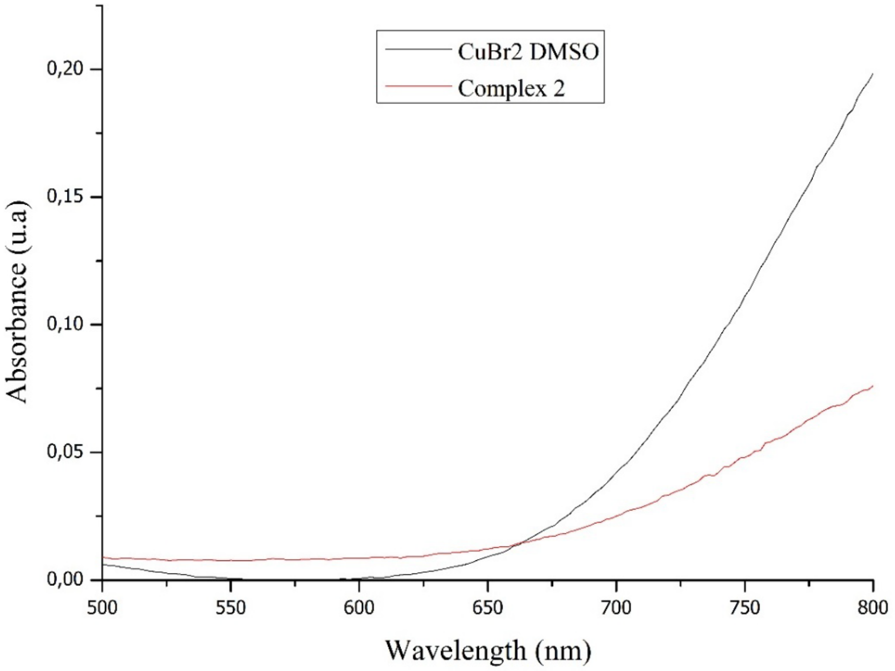

Figure S21: Comparison of the d–d transition observed for **Complex 2** (red) and the metal salt (black) at a concentration of 1×10^{-3} M, within the 500–800 nm range.

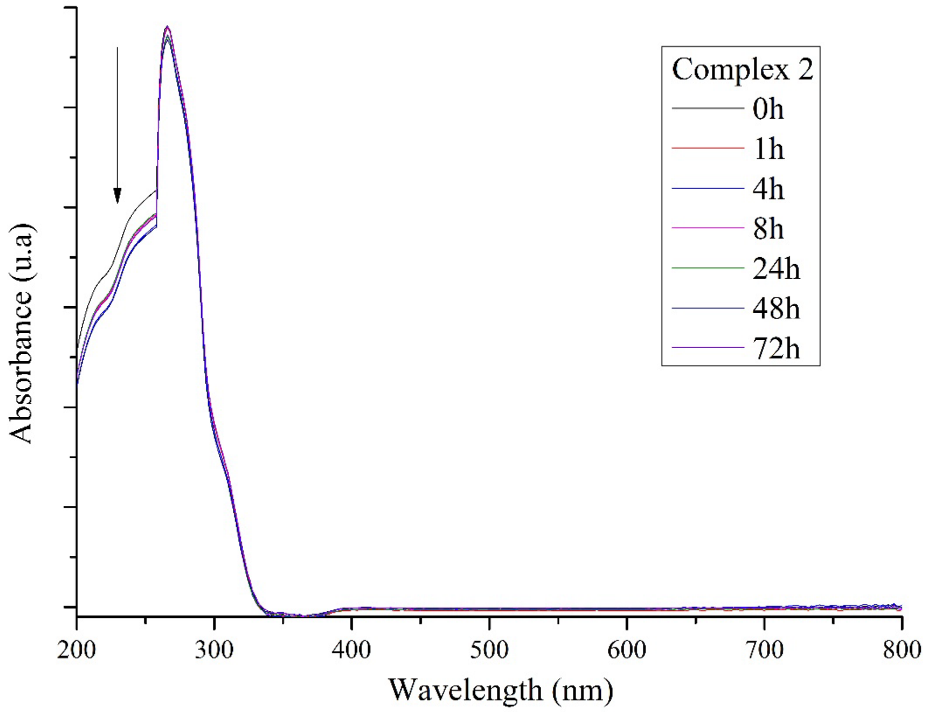

Figure S22: Time-dependent stability study of **Complex 2** in DMSO at a concentration of 1 × 10^{-5} M at 35°C.

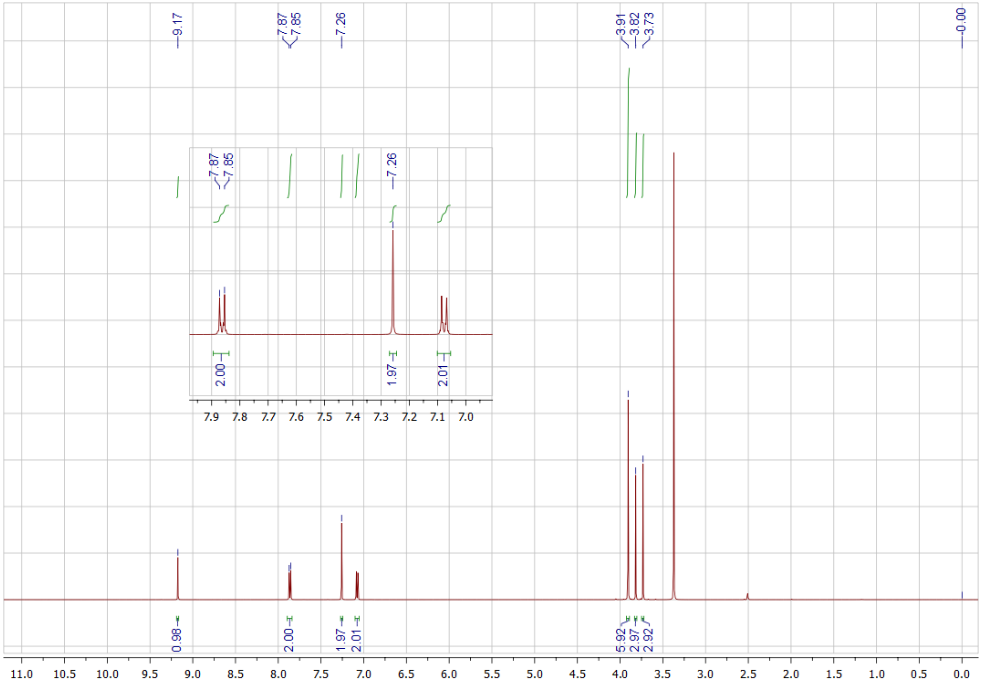

Figure S23: ^{1}H NMR spectrum of Ligand in DMSO-d_6 solution (δ in ppm).

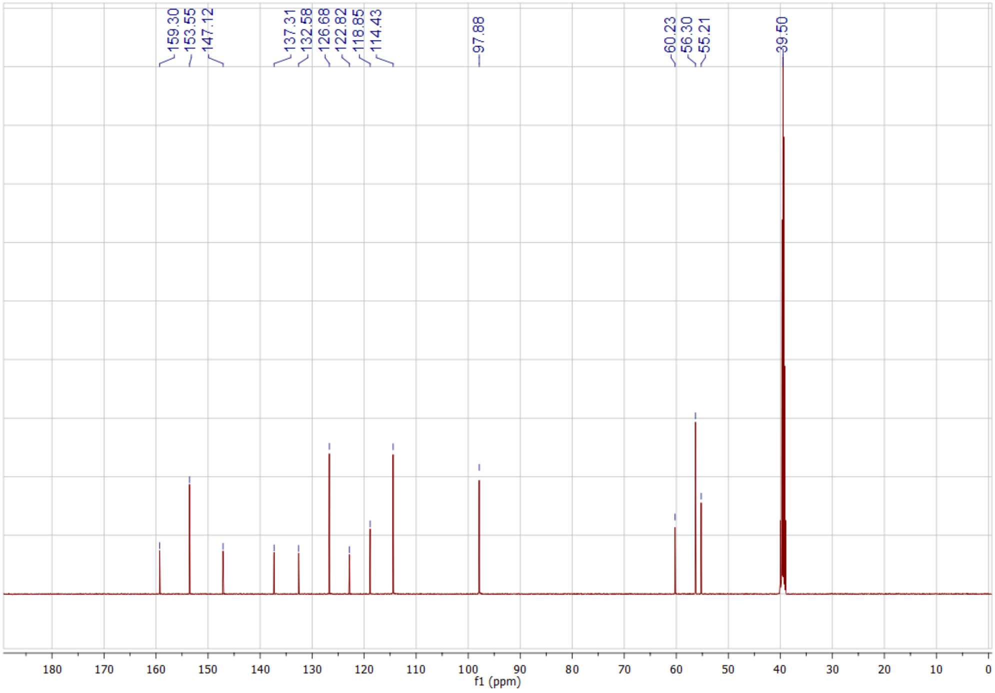

Figure S24: ^{13}C NMR spectrum of Ligand in DMSO-d_6 solution (δ in ppm).

Article	Structure	IC_{50} (µM)
Ana B. Caballero[8]	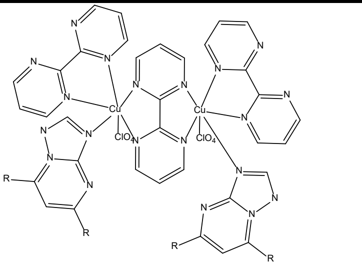	63.1 ± 5.7
Nedra Touj[9]	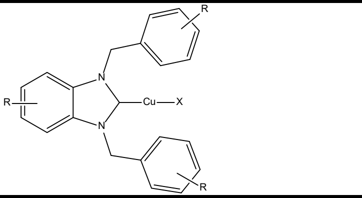	0.665 - 56.907
Ayse Yagmurlu[10]	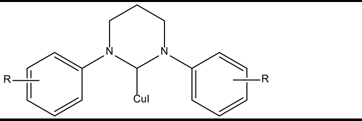	0.39 – 8.1
José Aleixo de Azevedo-França[11]	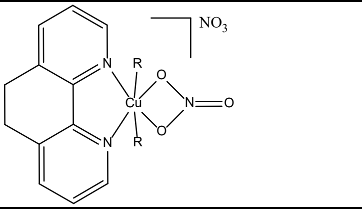	0.0534 – 0.1275
	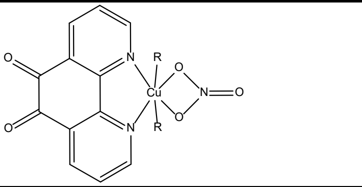	0.0149 – 1.5530
**Complex 1**	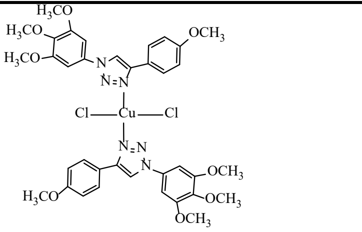	0.4± 0.0
**Complex 2**	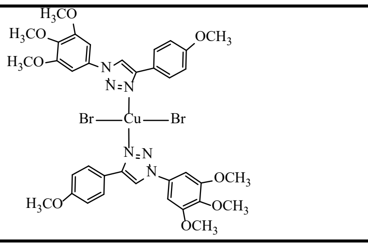	12.0±1.0

Table S7. IC_{50} values reported in the literature and for the studied complexes.

2. References

1. Sheldrick, G.M. A Short History of *SHELX*. *Acta Crystallogr A Found Crystallogr* **2008**, *64*, 112–122, doi:10.1107/S0108767307043930.
2. Pennington, W.T. ***DIAMOND* – Visual Crystal Structure Information System** . *J Appl Crystallogr* **1999**, *32*, 1028–1029, doi:10.1107/S0021889899011486.
3. Farrugia, L.J. *WinGX* and *ORTEP for Windows* : An Update. *J Appl Crystallogr* **2012**, *45*, 849–854, doi:10.1107/S0021889812029111.
4. Lee, C.; Yang, W.; Parr, R.G. Development of the Colle-Salvetti Correlation-Energy Formula into a Functional of the Electron Density. *Phys. Rev. B* **1988**, *37*, 785–789, doi:10.1103/PhysRevB.37.785.
5. Becke, A.D. Density-functional Thermochemistry. I. The Effect of the Exchange-only Gradient Correction. *The Journal of Chemical Physics* **1992**, *96*, 2155–2160, doi:10.1063/1.462066.
6. M. J. Frisch, G. W. Trucks, H. B. Schlegel, G. E. Scuseria, M. A. Robb, J. R. Cheeseman, G. Scalmani, V. Barone, G. A. Petersson, H. Nakatsuji, X. Li, M. Caricato, A. V. Marenich, J. Bloino, B. G. Janesko, R. Gomperts, B. Mennucci, H. P. Hratchian, J. V. Ortiz, A. F. Izmaylov, J. L. Sonnenberg, D. Williams-Young, F. Ding, F. Lipparini, F. Egidi, J. Goings, B. Peng, A. Petrone,; T. Henderson, D. Ranasinghe, V. G. Zakrzewski, J. Gao, N. Rega, G. Zheng, W. Liang, M. Hada, M. Ehara, K. Toyota, R. Fukuda, J. Hasegawa, M. Ishida, T. Nakajima, Y. Honda, O. Kitao, H. Nakai, T. Vreven, K. Throssell, J. A. Montgomery, Jr., J. E. Peralta, F. Ogliaro, M. J. Bearpark, J. J. Heyd, E. N. Brothers, K. N. Kudin, V. N. Staroverov, T. A. Keith, R. Kobayashi, J. Normand, K. Raghavachari, A. P. Rendell, J. C. Burant, S. S. Iyengar, J. Tomasi, M. Cossi, J. M. Millam, M. Klene, C. Adamo, R. Cammi, J. W. Ochterski, R. L. Martin, K. Morokuma, O. Farkas, J. B. Foresman, and D. J. Fox Gaussian 16 2016.
7. Hanwell, M.D.; Curtis, D.E.; Lonie, D.C.; Vandermeersch, T.; Zurek, E.; Hutchison, G.R. Avogadro: An Advanced Semantic Chemical Editor, Visualization, and Analysis Platform. *J Cheminform* **2012**, *4*, 17, doi:10.1186/1758-2946-4-17.
8. Caballero, A.B.; Marín, C.; Ramírez-Macías, I.; Rodríguez-Diéguez, A.; Quirós, M.; Salas, J.M.; Sánchez-Moreno, M. Structural Consequences of the Introduction of 2,2′-Bipyrimidine as Auxiliary Ligand in Triazolopyrimidine-Based Transition Metal Complexes. In Vitro Antiparasitic Activity. *Polyhedron* **2012**, *33*, 137–144, doi:10.1016/j.poly.2011.11.020.
9. Touj, N.; Nasr, I.S.A.; Koko, W.S.; Khan, T.A.; Özdemir, I.; Yasar, S.; Mansour, L.; Alresheedi, F.; Hamdi, N. Anticancer, Antimicrobial and Antiparasitical Activities of Copper(I) Complexes Based on *N* -Heterocyclic Carbene (NHC) Ligands Bearing Aryl Substituents. *Journal of Coordination Chemistry* **2020**, *73*, 2889–2905, doi:10.1080/00958972.2020.1836359.
10. Yagmurlu, A.; Buğday, N.; Yaşar, Ş.; Boulebd, H.; Mansour, L.; Koko, W.S.; Hamdi, N.; Yaşar, S. Synthesis, DFT Calculations, and Investigation of Catalytic and Biological Activities of Back-bond Functionalized re-NHC-Cu Complexes. *Applied Organom Chemis* **2024**, *38*, e7545, doi:10.1002/aoc.7545.
11. De Azevedo-França, J.A.; Feliciano Dos Santos Ramos, V.; Messori, L.; Santanni, F.; Sorace, L.; Pereira Borba-Santos, L.; Rozental, S.; Cola Fernandes Rodrigues, J.; Navarro, M. Synthesis, Characterization, and Biological Evaluation of Hybrid Copper(II) Complexes Containing Azole Drugs and Planar Ligands against Neglected Diseases. *New J. Chem.* **2024**, *48*, 2515–2526, doi:10.1039/D3NJ04608G.
